# Long noncoding RNA *BCRP3* stimulates VPS34 and autophagy activities to promote protein homeostasis and cell survival

**DOI:** 10.1186/s12929-022-00815-0

**Published:** 2022-05-10

**Authors:** Ruei-Liang Yan, Chiu-Lin Luan, Chun-Chieh Liao, Li-Heng Liu, Fei-Yun Chen, Hsin-Yi Chen, Ruey-Hwa Chen

**Affiliations:** 1 Institute of Biological Chemistry, Academia Sinica, Taipei, 115 Taiwan; 2 Institute of Molecular Medicine, College of Medicine, National Taiwan University, Taipei, 100 Taiwan; 3 Genome and Systems Biology Degree Program, College of Life Science, National Taiwan University, Taipei, 106 Taiwan; 4 Graduate Institute of Cancer Biology and Drug Discovery, College of Medical Science and Technology, Taipei Medical University, Taipei, 110 Taiwan; 5 Ph.D. Program for Cancer Molecular Biology and Drug Discovery, College of Medical Science and Technology, Taipei Medical University, Taipei, 110 Taiwan

**Keywords:** LncRNA, Autophagy, VPS34 complex, Protein quality control, TGF-β signaling, Cell death

## Abstract

**Background:**

Autophagy plays important roles in cell homeostasis and protein quality control. Long non-coding RNAs (lncRNAs) have been revealed as an emerging class of autophagy regulators, but the majority of them function in regulating the expression of autophagy-related genes. LncRNAs that directly act on the core autophagic proteins remain to be explored.

**Methods:**

Immunofluorescence staining and Western blotting were used to evaluate the function of *BCRP3* in autophagy and aggrephagy. RNA immunoprecipitation and in vitro RNA–protein binding assay were used to evaluate the interaction of *BCRP3* with its target proteins. Phosphatidylinositol 3-phosphate ELISA assay was used to quantify the enzymatic activity of VPS34 complex. qRT-PCR analysis was used to determine *BCRP3* expression under stresses, whereas mass spectrometry and Gene Ontology analyses were employed to evaluate the effect of *BCRP3* deficiency on proteome changes.

**Results:**

We identified lncRNA *BCRP3* as a positive regulator of autophagy. *BCRP3* was mainly localized in the cytoplasm and bound VPS34 complex to increase its enzymatic activity. In response to proteotoxicity induced by proteasome inhibition or oxidative stress, *BCRP3* was upregulated to promote aggrephagy, thereby facilitating the clearance of ubiquitinated protein aggregates. Proteomics analysis revealed that *BCRP3* deficiency under proteotoxicity resulted in a preferential accumulation of proteins acting in growth inhibition, cell death, apoptosis, and Smad signaling. Accordingly, *BCRP3* deficiency in proteotoxic cells compromised cell proliferation and survival, which was mediated in part through the upregulation of TGF-β/Smad2 pathway.

**Conclusions:**

Our study identifies *BCRP3* as an RNA activator of the VPS34 complex and a key role of *BCRP3*-mediated aggrephagy in protein quality control and selective degradation of growth and survival inhibitors to maintain cell fitness.

**Supplementary Information:**

The online version contains supplementary material available at 10.1186/s12929-022-00815-0.

## Background

The removal of ubiquitinated protein aggregates is crucial for cell homeostasis and its impairment is associated with many pathological conditions, such as aging and neurodegenerative disorders [1, 2]. Although proteasome represents the main route for degradation of polyubiquitinated proteins, ubiquitinated protein aggregates are often resistant to proteasome degradation and are processed by macroautophagy (thereafter referred to as autophagy) [3, 4]. Autophagy is characterized by the formation of double-membrane vesicle structures in the cytoplasm, called autophagosomes. Cellular components are sequestered in the autophagosomes and subsequently degraded when autophagosomes fuse with lysosomes. The formation of double-membrane autophagosome requires the sequential actions of a number of ATG proteins [5]. Among them, the VPS34 complex, formed by class III phosphatidylinositol-3-kinase (PI3K) VPS34, together with the accessory proteins Beclin 1, VPS15, ATG14, and AMBRA1, is activated by the upstream kinases ULK1/2 and is responsible for generating phosphatidylinositol 3-phosphate (PI3P) in the nascent phagopores to promote the nucleation of autophagosomes [6]. Consistent with its important role in an early step of the autophagosome biogenesis process, this complex serves as a hub to integrate various signals and regulators that impact positively or negatively on the autophagy process [7].

The autophagy process for selective degradation of ubiquitinated substrates is called aggrephagy [8]. Similar to other types of selective autophagy, cargo selection is dependent on a set of cargo receptors the link cargos to the autophagic machinery. The major cargo receptor for aggrephagy is p62/SQSTM1 [9], although NBR1, Tollip, Optineurin, and TAXBP1 also participate in this process [10–13]. Notably, p62 controls both the assembly and degradation of protein aggregates. That is, the binding of polyubiquitinated proteins to the oligomeric p62 triggers a phase separation to concentrate the ubiquitinated proteins into larger condensates, where p62 facilitates the tethering of ubiquitin-positive condensates to the nascent autophagosome membrane through its LC3-interacting region (LIR) [9, 14–18]. The ATG8 family proteins LC3B and GABARAP are the first identified partners of p62. In addition, a recent study revealed the interaction of p62 LIR with FIP200, a subunit of the ULK1/2 complex, and that FIP200 and ATG8 family proteins compete for binding p62 [19]. Thus, the current model of aggrephagy indicates that p62 governs a sequential recruitment of upstream ATG proteins, such as ULK1 complex, VPS34 complex and ATG16L1, followed by a replacement of the ULK1 complex with the ATG8 family proteins for autophagosome expansion. Accordingly, blockage of VPS34 activity impairs aggrephagy to impede the clearance of ubiquitinated protein aggregates [20, 21].

LncRNAs, which comprise the largest part of mammalian transcriptome, have been found to participate in diverse cellular processes to influence on cell fitness. LncRNAs exert cellular effects by forming RNA–DNA, RNA–RNA or RNA–protein complex and this biochemical versatility makes mechanistic analysis rather challenging [22]. Recent studies have indicated lncRNAs as an emerging class of autophagy regulators and lncRNA-mediated regulations occur in various steps of the autophagosome formation and maturation process [23–25]. However, most lncRNAs function in regulating the expression of autophagic genes through mechanisms involving miRNAs [24, 25]. LncRNAs that bind and alter the activity/function of autophagic proteins have been scarcely reported, although lncRNA *NBR2* fits this category by binding and activating AMPK for upregulating autophagy activity [26]. Here, we report lncRNA *break point region pseudogene 3 (BCRP3)* (https://www.ncbi.nlm.nih.gov/gene/?term=BCRP3) as an autophagy-stimulating factor by binding and activating VPS34 complex. The expression of *BCRP3* is upregulated in response to proteotoxicity induced by proteasome inhibition and oxidative stress, thus facilitating an enhanced aggrephagy activity. We provide evidence that this mechanism is important in protein quality control and cell survival.

## Methods

### Plasmids

*BCRP3* cDNA was amplified from the genomic DNA of BT474 cells using the primers: Fw: 5′ CCGGAATTCACTCCGTAGTGTGCACTTGGT and Rv: 5′ACGCGTCGACTTTTCGTCTCAGGAAACTTTTTAATG. The cDNA was subcloned to the lentiviral vector pLAS5w.Pneo. *BCRP3* shRNAs were predicted via BLOCK-iT™ RNAi Designer (https://rnaidesigner.thermofisher.com/), generated by Purigo Biotechnology, Taipei, Taiwan, and cloned to pLKO.1. The shRNA targeting sequences are as follows: *shLuc* 5′TTACGCTGAGTACTTCGA; *shBCRP3#1* 5′ATATTGGACGCTGGACCCGC; *shBCRP3#2* 5′ GACGCTGGACCCGCAGGCCC. Plasmids encoding EGFP-2xFYVE, HA-Beclin 1, and FLAG-Beclin 1 were kindly provided by Guang-Chao Chen (Academia Sinica, Taipei, Taiwan). Plasmids encoding GFP-DFCP1, GFP-LC3, and RFP-p62 were kindly provided by Wei Yuan Yang (Academia Sinica). pcDNA4-VPS34-FLAG was purchased from Addgene, Watertown, MA, USA (#24398). The 4 × SBE-Luc construct was obtained from Rik Derynck (University of California at San Francisco).

### Cell culture and transfection

HCT116 cells were maintained in Roswell Park Memorial Institute (RPMI) 1640 medium with 10% fetal bovine serum (FBS) and 1% penicillin/streptomycin (PS). HeLa cells were cultured in Minimum Essential Medium (MEM) supplemented with 10% FBS, 1% PS, and 1 mM sodium pyruvate. *BCRP3*-deficient cells were generated by lentiviral transduction of *BCRP3* shRNAs. For starvation, cells were incubated with Earle's Balanced Salt Solution (EBSS). Plasmid transfection was performed using Lipofectamine 2000 reagent (Invitrogen, Thermo Fisher Scientific, Waltham, MA, USA) according to the manufacturer’s instructions.

### Lentiviral package and transduction

For lentiviral production, 293FT cells were co-transfected with package plasmid (pCMV8.91), envelop VSV-G plasmid (pMD.G), and *BCRP3* cDNA or shRNA expressing plasmid at a weight ratio of 4:1:4. After 48 h of incubation, supernatant containing lentivirus was harvested and filtered through a 0.22 μm syringe filter. For *BCRP3* knockdown, HeLa and HEK293T cells at 70% confluency were transduced with lentivirus carrying *BCRP3*-specific shRNA in polybrene (8 μg/ml)-containing medium, and the transduced cells were selected with 1 μg/ml of puromycin (Sigma-Aldrich, St Louis, MO, USA). For *BCRP3* overexpression, HeLa and HCT116 cells were transduced with lentivirus carrying pLAS5w.Pneo-*BCRP3* and selected with 0.5 mg/ml G418 geneticin (Invitrogen, Thermo Fisher Scientific).

### Antibodies and reagents

Antibodies used in this study were obtained from commercial sources and described in Additional file 1: Table S1. TβRI (type I TGF-β receptor) inhibitor SB431542, 5-fluorouracil (5-FU), and 3-methyladenine (3-MA) were from Sigma-Aldrich. MG132 was from Calbiochem, San Diego, CA, USA. As_2_O_3_ was a gift from Hsiu-Ming Shih (Academia Sinica).

### Western blotting

Cells were lysed with NP-40 lysis buffer containing 150 mM NaCl, 50 mM Tris–HCl pH 7.5, 1% NP-40, 1 mM PMSF, and protease inhibitor cocktail (Roche, Indianapolis, IN, USA). The lysates were clarified by centrifugation at   15,490 × g for 15 min. The protein concentration was determined with Bradford reagent. Lysates with an equal amount of proteins were boiled with sample buffer, resolved by SDS-PAGE, and then transferred to PVDF membrane. After blocked with 5% non-fat milk and probed with the primary antibodies and subsequently with the secondary antibodies, membranes were proceeded for signal development using the Western Lightning Plus-ECL reagent (PerkinElmer, Waltham, MA, USA).

### Immunoprecipitation

Cells were lysed with NP-40 lysis buffer or RIPA lysis buffer (150 mM NaCl, 20 mM Tris–HCl pH 7.5, 1% NP-40, 0.1% SDS, 1 mM PMSF, and protease inhibitor cocktail). After centrifugation at 15,490 × g, the supernatant was pre-cleared with protein A beads for 1 h, followed by incubation with the desired antibodies for 1.5 h. The beads were washed three times, denatured with sample buffer, and proceeded for Western blotting analysis. For FLAG or GFP immunoprecipitation, anti-FLAG agarose beads (M2; Merck Millipore, Billerica, MA, USA) or GFP-Trap agarose beads (ChromoTek, Hauppauge, NY, USA) were used.

### Immunofluorescence

Cells grown on coverslips in a 6-well plate were fixed with 4% paraformaldehyde at room temperature for 15 min, followed by permeabilization with chilled methanol on ice for 10 min. Cells were then blocked with 1% BSA and 10% goat serum in PBS for 1 h, followed by incubation with primary antibodies at 4℃ for overnight. Next, fluorescence-tagged secondary antibodies were added and incubated at room temperature for 1 h together with DAPI for nuclear staining. Samples were mounted on glass slides using fluorescence mounting medium (DAKO, Carpinteria, CA, USA) and examined under an Olympus FV3000 confocal microscope (Olympus Co., Tokyo, Japan) with 60x/1.40 oil objective lens. For images analysis and cell counting, the ImageJ software (https://imagej.nih.gov/ij/download.html) was used.

### Quantitative real-time PCR (qRT-PCR)

Total RNA was isolated from cells with Trizol reagent (Invitrogen, Thermo Fisher Scientific). 1 μg of RNA per sample was subjected to reverse transcription using the iScript cDNA Synthesis kit (Bio-Red, Richmond, CA, USA) according to the manufacturer’s protocol. qPCR was performed with the FastStart Universal SYBR Green Master reagent (Roche) on a LightCycler® 480 instrument II system (Roche). *GAPDH* was used as a reference gene. The sequences of PCR primers are listed in Additional file 1: Table S2.

### Subcellular fractionation assay

1 × 10^7^ cells were lysed in 10 ml cytoplasmic lysis buffer (0.256 M sucrose, 8 mM Tris–HCl pH 7.5, 4 mM MgCl_2_, 0.8% Triton X-100, and 0.25% PBS) with gentle pipetting and inverting, followed by rotation at 4℃ for 10 min and incubation on ice for another 10 min. After centrifugation at 2,500 × g for 15 min, 250 μl (1/40 volume) of supernatant was transferred to a new tube and saved as the cytoplasmic fraction. The pellet was washed with 10 ml ice cold PBS twice and then lysed with 1 ml RIP buffer (50 mM Tris–HCl pH 7.4, 150 mM NaCl, 1 mM EDTA, 1% NP-40 and 0.5 mM dithiothreitol, 1:100 SuperRNaseIn (Invitrogen, Thermo Fisher Scientific), and protease inhibitor cocktail). The lysate was sonicated using Qsonica (30 s ON, 30 s OFF for 15 min) and centrifuged at 13,000 rpm for 10 min. The supernatant was collected as the nuclear fraction. An equal portion (1/40 volume) of the cytoplasmic and nuclear fractions was subjected to RNA extraction with 1 ml Trizol reagent.

### RNA fluorescence in situ hybridization (FISH)

RNA FISH was carried out using the BaseScope Assay kit (Advanced Cell Diagnostics, Newark, CA, USA) according to the manufacturer’s protocol. Briefly, HeLa cells cultured on slides were fixed with 4% paraformaldehyde at room temperature for 15 min, treated with hydrogen peroxide at room temperature for 10 min, and then digested with Protease III (1:15 dilution) at room temperature for 10 min. *BCRP3*-specific probes were custom produced by Advanced Cell Diagnostics (Cat#1055141-C1) and incubated with cells at 40℃ for 2 h in a HybEZ™ Oven (Advanced Cell Diagnostics), followed by multistep signal amplification as instructed in the protocol. Finally, cells were mounted and imaged using an Olympus FV3000 confocal microscope with 60x/1.40 oil objective lens.

### RNA immunoprecipitation

RNA immunoprecipitation was followed by a protocol described previously [27]. Briefly, cells were lysed with RIP buffer, kept on ice for 1 h, and then centrifuged at 14,000 rpm for 10 min at 4 ℃. The supernatant was pre-cleared with protein A beads for 1 h at 4 ℃ and then incubated with various antibodies for 2 h at 4 ℃. The RNA-associated immunocomplexes were then captured by protein A beads for 1 h at 4℃. After washes, the beads were resuspended in 100 μl lysis buffer. 10 μl (1/10 volume) of resuspended mixture was saved for Western blotting analysis and the remaining 90 μl (9/10 volume) were proceeded for RNA extraction with Trizol reagent, followed by qRT-PCR analysis.

### In vitro RNA–protein binding assay

The full-length *BCRP3* cDNA was cloned to pcDNA3.1 in a sense direction. Biotin-labeled *BCRP3* was synthesized by in vitro transcription using RNA Labeling Mix (Roche) and T7 RNA polymerase (Ambion, Austin, TX, USA), and purified by NucleoSpin® RNA Isolation kit (Macherey–Nagel, Bethlehem, PA, USA). Biotinylated *BCRP3* was folded in RNA structure buffer (10 mM Tris–HCl pH 7.0, 100 mM KCl, and 10 mM MgCl_2_) for 2 min at 90℃, immediately chilled on ice for 2 min, and incubated at room temperature for 20 min to form the proper secondary structure. GFP-ATG14 plasmid was transfected into 293T cells and 1.5 mg cell lysates were immunoprecipitated with 80 μl 50% slurry of GFP-TRAP beads. The ATG14 immunocomplexes were then incubated with in vitro synthesized folded *BCRP3* (2 pmol) for 1 h at 4℃. After washes, the beads were divided into two aliquots. One of the aliquots was used for Western blotting while the other was subjected to RNA extraction with Trizol reagent, followed by qRT-PCR analysis.

### PI3K activity assay

The FLAG-VPS34 complex was immunoprecipitated from HeLa cells and subjected to activity assay using the Class III PI3K ELISA Kit (K-3000, Echelon Biosciences, Salt Lake City, UT, USA). Briefly, 30 μl kinase reaction buffer (10 mM Tris–HCl pH 8.0, 100 mM NaCl, 1 mM EDTA, 10 mM MnCl_2_, and 50 μM ATP), 9 μl of 500 μM PI substrate, and 20 μl ddH_2_O were added to the immunocomplex, together with the in vitro transcribed *BCRP3* or control RNA. After incubation at 30℃ for 30 min, the reaction was terminated by adding 12 μl 100 mM EDTA. The amount of PI3P produced was determined by a competitive ELISA assay. The quenched reaction was diluted and added to the PI3P-coated plate for competitive binding to the PI3P detector. The amount of PI3P was detected by reading the colorimetric changes at the absorbance of 450 nm. The concentration of PI3P was calculated as inversely proportional to the ELISA signal, and normalized to the amount of immunoprecipitated FLAG-VPS34.

### Luciferase assay

Luciferase assay was conducted with the Dual-Glo Luciferase Reporter Assay System (Promega, Madison, WI, USA) followed by the manufacturer’s instructions. The Firefly luciferase activity was normalized to that of Renilla luciferase activity.

### Apoptosis assay

Apoptotic cell death was measured using the Cell Death Detection ELISA plus kit (Roche) according to the manufacturer’s instructions. Briefly, 3 × 10^5^ cells were plated on 6 cm dishes for various treatments and were lysed using 250 μl lysis buffer. After centrifugation, 20 μl of supernatant was added onto the streptavidin-coated plate together with 80 μl Immunoreagent (biotin-conjugated anti-histone antibody and peroxidase-conjugated anti-DNA antibody) and the mixture was incubated for 2 h at 25 ℃. Then, 100 μl of ABTS substrate solution was added to each well and the colorimetric changes were monitored at the absorbance of 405 nm.

### BrdU incorporation and MTT assays

For assaying BrdU incorporation, cells were seeded on 96-well plates at a density of 2000 cells/well, cultured overnight, and treated with various inhibitors. Then, cells were pulse labeled with 10 μM BrdU for 24 h. After fixation, BrdU incorporation was determined by the BrdU Cell Proliferation Assay kit (Merck Millipore) according to the manufacturer's instructions. For MTT assay, cells seeded on 96-well plate at a density of 1 × 10^4^ cells per well were treated with various agents, followed by the addition of MTT solution (5 mg/ml). Next, the medium was removed and cells were lysed by DMSO. Cell viability was determined by absorbance measurement at 570 nm.

### Sample preparation for liquid chromatography-tandem mass spectrometry (LC–MS/MS)

1 × 10^7^ HeLa cells were lysed in denaturing buffer (8 M urea and 20 mM HEPES pH 8.0), sonicated, and centrifuged at 13,000 rpm for 15 min at 25℃. An aliquot of 5 μg protein was reduced with 10 mM dithioerythritol at 37 °C for 1 h, and then alkylated with 25 mM iodoacetamide in the dark for 1 h. The protein sample was digested with Lys-C/trypsin for 19 h and terminated with formic acid at a final concentration of 0.1%. The digested sample was desalted by Zip-Tip and lyophilized prior to LC–MS/MS analysis.

### Shotgun proteomic identifications

NanoLC−nanoESi-MS/MS analysis was performed on a Thermo UltiMate 3000 RSLCnano system connected to a Thermo Orbitrap Fusion mass spectrometer (Thermo Fisher Scientific, Bremen, Germany) equipped with a nanospray interface (New Objective, Woburn, MA, USA) and followed procedures as described previously [28]. Peptide mixtures were loaded onto a 75 μm ID, 25 cm length PepMap C18 column (Thermo Fisher Scientific) packed with 2 μm particles with a pore width of 100 Å and were separated using a segmented gradient in 120 min from 5 to 35% solvent B (0.1% formic acid in acetonitrile) at a flow rate of 300 nl/min. Solvent A was 0.1% formic acid in water. The mass spectrometer was operated in the data-dependent mode. Briefly, survey scans of peptide precursors from 350 to 1600 m/z were performed at 240 K resolution with a 2 × 10^5^ ion count target. Tandem MS was performed by isolation window at 1.6 Da with the quadrupole, higher-energy collisional dissociation fragmentation with normalized collision energy of 30, and rapid scan MS analysis in the ion trap. The MS^2^ ion count target was set to 1 × 10^4^ and the max injection time was 50 ms. Only those precursors with charge state 2–6 were sampled for MS^2^. The instrument was run in top speed mode with 3 s cycles; the dynamic exclusion duration was set to 15 s with a 10 ppm tolerance around the selected precursor and its isotopes. Monoisotopic precursor selection was turned on.

Peptide identification was performed using the percolator node within Proteome Discoverer (v 2.4.1.15; Thermo Scientific, Waltham, MA, USA) against the Swiss-Prot Human database (561,911 entries total). Search criteria used were trypsin digestion, variable modifications set as carbamidomethyl (C), oxidation (M), ubiquitinylation (K) allowing up to two missed cleavages, mass accuracy of 10 ppm for the parent ion and 0.02 Da for the fragment ions. The false discovery rate (FDR) was set to 1% for peptide identifications. For label-free quantification, precursor ions intensities were extracted using Minora Feature Detector node in Proteome Discoverer with a 2 ppm mass precision and 2 min retention time shift.

### Bioinformatics

For in silico analysis of *BCRP3* expression, RNA-seq data of different types of normal and cancer tissues were downloaded from The Cancer Genome Atlas (TCGA) or Genotype-Tissue expression (GTEx) database via the UCSC Xena platform (http://xena.ucsc.edu) [29]. Gene Ontology (GO) analysis was carried out by DAVID (https://david.ncifcrf.gov/), and filtered by *P* value < 0.05.

### Statistics

The unpaired two sided Student’s t-test was used to compare between two groups and one-way or two-way ANOVA with Tukey’s post hoc test was used for multi-group comparisons.

## Results

### *BCRP3* promotes autophagy

**Fig. 1:**
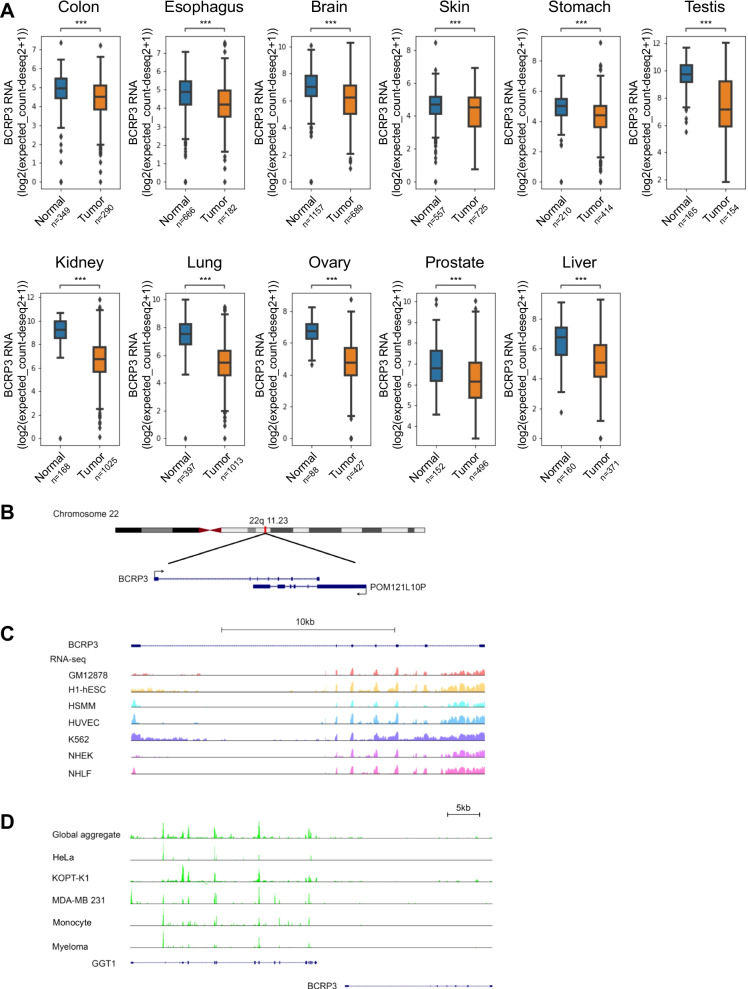
Characterization of the *BCRP3* gene expression and gene locus. **A** *BCRP3* expression in tumor and normal tissues of indicated cancer types retrieved from TCGA data sets. Whiskers indicate min to max, bounds of box correspond to the fist and third quartiles and the center line represents the median. *P* values are determined by unpaired t-test, ***P < 0.001. **B** Genomic loci for lncRNAs *BCRP3* and *POM121L10P*. Exons and the direction of transcription are indicated by filled boxes and arrows, respectively. **C** *BCRP3* RNA-seq data from indicated cells were retrieved from the UCSC database. **D** Ribo-seq data on *BCRP3* and its neighboring protein-coding gene *GGT1* from indicated cells were retrieved from the UCSC database

**Fig. 2:**
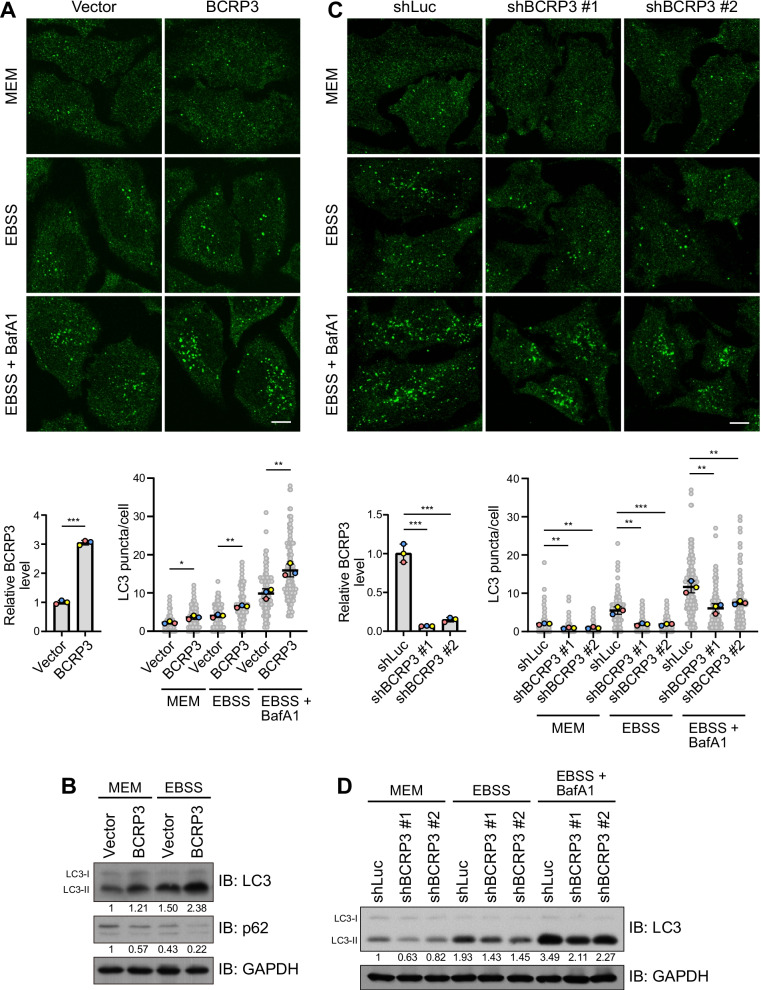
*BCRP3* promotes autophagosome formation**. A, C** Immunofluorescence staining of LC3 in HeLa cells stably expressing vector or *BCRP3* (**A**) or Luc or *BCRP3* shRNAs (**C**) and cultured in full medium (MEM) or EBSS together with or without 200 nM bafilomycin A1 for 2 h. Representative confocal images are shown on the top and quantitative data are on the bottom right. Bar, 10 μm. Relative expression level of *BCRP3* was analyzed by qRT-PCR and shown on the bottom left. Data are means ± SD from three independent experiments and 30 cells per group per experiment were counted. *P* values are determined by unpaired t-test (**A**) or one-way ANOVA with Tukey’s post hoc test (**C**), *P < 0.05, **P < 0.01, ***P < 0.001. **B, D** Western blot analysis of indicated proteins in HeLa cells generated and treated as in (**A**) or (**C**), respectively. The levels of LC3-II and p62 are normalized with that of GAPDH and shown on the bottom

By analyzing TCGA data sets, we identified lncRNA *BCRP3* based on its lower expression in tumor than normal tissues in many cancer types, including colon, esophagus, brain, skin, stomach, testis, kidney, lung, ovary, prostate, and liver (Fig. 1A). The *BCRP3* gene is located on the chromosome 22q11.23 and is overlapping in an antisense orientation with the gene of lncRNA *POM121L10P* (Fig. 1B). RNA-seq data retrieved from the GTEx database revealed the expression of *BCRP3* in many cell types and tissues (Fig. 1C and Additional file 1: Fig. S1). However, Ribo-seq data indicated the devoid of ribosome binding to *BCRP3*, which was in a sharp contrast to the RNA of its neighboring coding gene *GGT1* (Fig. 1D). Accordingly, *BCRP3* is denoted by National Center for Biotechnology Information (NCBI) GenBank as a pseudogene which gives rise to a lncRNA of 1.4 kb. The downregulation of *BCRP3* in many cancer types suggested its role in tumor suppression. We therefore tested the functions of *BCRP3* in cancer. While overexpression of *BCRP3* in HCT116 human colorectal cancer cell line did not affect cell viability at the basal state, it significantly increased cell viability upon treatment with a chemotherapeutic drug 5-FU (Additional file 1: Fig. S2A). Monitoring DNA damage by γH2A.X showed no difference between control and *BCRP3*-overexpressed cells at each time point after 5-FU treatment, suggesting that *BCRP3* does not affect DNA damage sensing/repair (Additional file 1: Fig. S2B). Notably, autophagy represents one mechanism that leads to the resistance of cancer cells to chemotherapy, but in contrast plays a suppressive role for tumor initiation [30, 31]. The latter is in line with the lower expression of *BCRP3* in tumor than normal tissues. Furthermore, we found that autophagy blockage by 3-MA or bafilomycin A1 diminished the chemoresistance effect of *BCRP3* (Additional file 1: Fig. S2C). We thus explored the role of *BCRP3* in autophagy. Remarkably, overexpression of *BCRP3* in HCT116 cells increased the number of autophagosomes (detected by LC3 puncta) in cells cultured in MEM (for measuring basal autophagy activity) or EBSS (for monitoring starvation-induced autophagy activity) (Additional file 1: Fig. S3A). A similar finding was observed in HeLa cells overexpressing *BCRP3* (Fig. 2A). Importantly, the increased autophagosome number by *BCRP3* overexpression was also evident in cells treated with bafilomycin A1 to block autophagic turnover (Fig. 2A), indicating that *BCRP3* promotes autophagosome formation rather than inhibiting autophagosome fusion with lysosome for degradation. Western blot analysis showed that *BCRP3* overexpression increased LC3 lipidation (designated as LC3-II) and decreased the abundance of p62, an autophagic cargo (Fig. 2B), further supporting an enhancement of autophagy activity. In the reciprocal set of experiments, we depleted *BCRP3* expression in HeLa or 293T cells by shRNAs. Importantly, *BCRP3* knockdown decreased autophagosome numbers and LC3 lipidation in cells cultured in MEM, EBSS, MEM with bafilomycin A1, or EBSS with bafilomycin A1 (Fig. 2C, D and Additional file 1: Fig. S3B, C). These findings collectively identify a role of *BCRP3* in promoting autophagosome biogenesis.

### *BCRP3* acts downstream of ULK1 and upstream of PI3P production

**Fig. 3:**
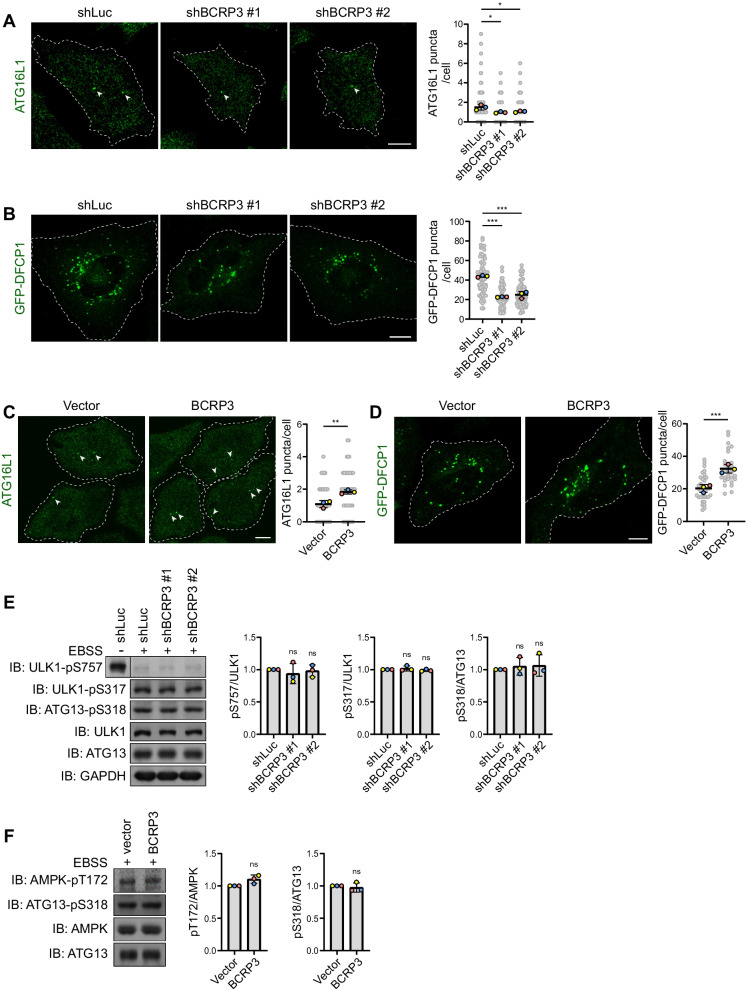
*BCRP3* promotes PI3P production but does not affect TORC1, AMPK, and ULK1 activities. **A, C** Immunofluorescence staining of ATG16L1 in control or *BCRP3*-deficient HeLa cells (**A**) or HeLa cells stably expressing vector or *BCRP3* (**C**) and starved in EBSS for 2 h. Representative confocal images are shown on the left and quantitative data are on the right. Arrowheads indicate the ATG16L1 puncta. Bar, 10 μm. Data are means ± SD from three independent experiments and 30 cells per group per experiment were counted. *P* values are determined by one-way ANOVA with Tukey’s post hoc test (**A**) or unpaired t-test (**C**), *P < 0.05, **P < 0.01. **B, D** Confocal microscopy analysis of HeLa cells derivatives as in (**A**) or (**C**), respectively, transiently transfected with GFP-DFCP1, and starved in EBSS for 2 h. Representative images are shown on the left and quantitative data are on the right. Bar, 10 μm. Data are means ± SD from three independent experiments and 20 (**B**) or 10 (**D**) cells per group per experiment were counted. *P* values are determined by one-way ANOVA with Tukey’s post hoc test (**B**) or unpaired t-test (**D**), ***P < 0.001. **E, F** Western blot analysis of indicated proteins in control or *BCRP3*-deficient HeLa cells (**E**) or HeLa cells stably expressing vector or *BCRP3* (**F**) and starved in EBSS for 1 h. The blots are representative of n = 3 independent experiments. Quantitative data are shown on the right, ns, not significant by one-way ANOVA with Tukey’s post hoc test (**E**) or unpaired t-test (**F**)

Next, we investigated the functional position of *BCRP3* in the autophagosome biogenesis process. ATG16L1 and WIPI2 are two proteins recruited to the PI3P-containing phagophore, a nascent autophagosomal structure, to facilitate LC3 lipidation [32, 33]. Notably, *BCRP3* knockdown decreased the formation of ATG16L1 and WIPI2 puncta in EBSS-cultured cells (Fig. 3A and Additional file 1: Fig. S4). Furthermore, *BCRP3* knockdown in EBSS-cultured cells diminished GFP-DFCP1 puncta (also known as omegasomes, PI3P-enriched compartments serving as the precursors of autophagosomes) [34] (Fig. 3B). In the reciprocal set of experiments, *BCRP3* overexpression in EBSS-cultured cells increased ATG16L1 puncta and GFP-DFCP1 puncta (Fig. 3C, D). However, *BCRP3* knockdown did not affect the kinase activities of TORC1, AMPK, and ULK1 in EBSS-cultured cells, as monitored by ULK1 S757 phosphorylation, ULK1 S317 phosphorylation, and ATG13 S318 phosphorylation, respectively [35, 36] (Fig. 3E). Likewise, *BCRP3* overexpression did not alter AMPK and ULK1 activities in cells cultured in EBSS (Fig. 3F). Since TORC1, AMPK, and ULK1 act upstream of VPS34 complex in the autophagosome biogenesis process, our data suggest an impact of *BCRP3* on the VPS34 complex.

### *BCRP3* binds VPS34 complex to increase its enzymatic activity

**Fig. 4:**
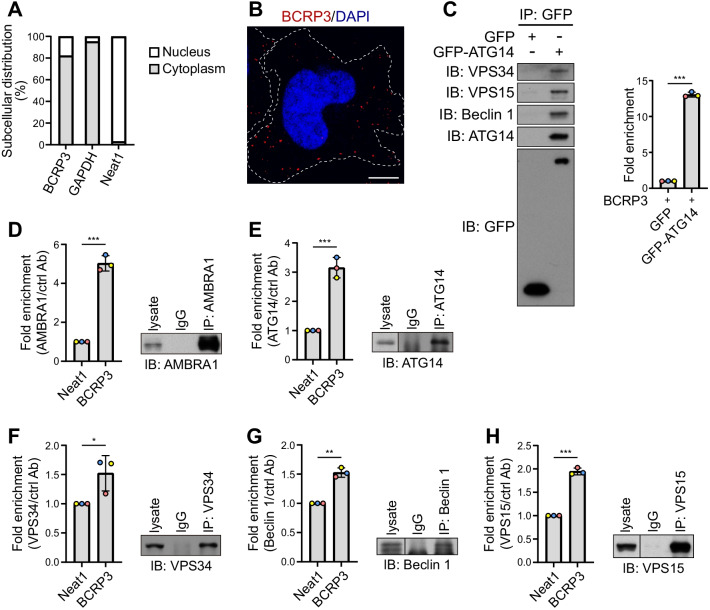
*BCRP3* is mainly distributed to the cytoplasm and binds VPS34 complex.** A** Subcellular localization of *BCRP3* in HeLa cells detected by cell fractionation followed by qRT-PCR analysis. *GAPDH* and *Neat1* were used as cytoplasmic and nuclear RNA controls, respectively. **B** RNA FISH analysis of *BCRP3* (red) in HeLa cells. Nuclei were stained with DAPI (blue). Bar, 10 μm. **C** In vitro RNA protein binding assay. GFP-ATG14 transiently expressed in 293T cells was immunoprecipitated with GFP-Trap beads and the immunocomplexes were incubated with in vitro transcribed *BCRP3*. The bound RNAs were extracted and analyzed by qRT-PCR, whereas the presence of VPS34 complex components in the immunocomplexes was analyzed by Western blot (left). Data are means ± SD from three independent experiments. *P* values are determined by unpaired t-test, ***P < 0.001. **D-H** RNA immunoprecipitation assay using indicated antibodies followed by qRT-PCR analysis. Data were normalized to the IgG control and expressed as means ± SD, n = 3. *P* values are determined by unpaired t-test, *P < 0.05, **P < 0.01, ***P < 0.001

To determine the mechanism by which *BCRP3* regulates the function of VPS34 complex, we first interrogated the subcellular localization of *BCRP3*. Cell fractionation followed by qRT-PCR analysis found that *BCRP3* is mainly present in the cytoplasm (Fig. 4A). This finding was further confirmed by RNA FISH analysis with a *BCRP3* antisense probe (Fig. 4B). Thus, we examined whether *BCRP3* acts directly on the cytoplasmic-residing VPS34 complex. Western blot analysis showed that *BCRP3* knockdown did not alter the abundance of VPS34 complex subunits, including VPS34, Beclin 1, VPS15, ATG14, and AMBRA1 (Additional file 1: Fig. S5A). Furthermore, immunoprecipitation analyses revealed that *BCRP3* knockdown did not compromise the integrity of VPS34 complex as well as the oligomerization of Beclin 1 (Additional file 1: Fig. S5A, B), the latter of which is crucial for the assembly of VPS34 complex [37]. We isolated VPS34 complex from cells transfected with GFP-ATG14 using GFP-Trap. Remarkably, this complex was readily associated with in vitro transcribed *BCRP3* (Fig. 4C), suggesting a direct binding between *BCRP3* and VPS34 complex. Furthermore, RNA immunoprecipitation analysis demonstrated the association of *BCRP3* with each of the VPS34 complex component in vivo (Fig. 4D–H). Thus, these data identify *BCRP3* as a binding partner of the VPS34 complex.

**Fig. 5:**
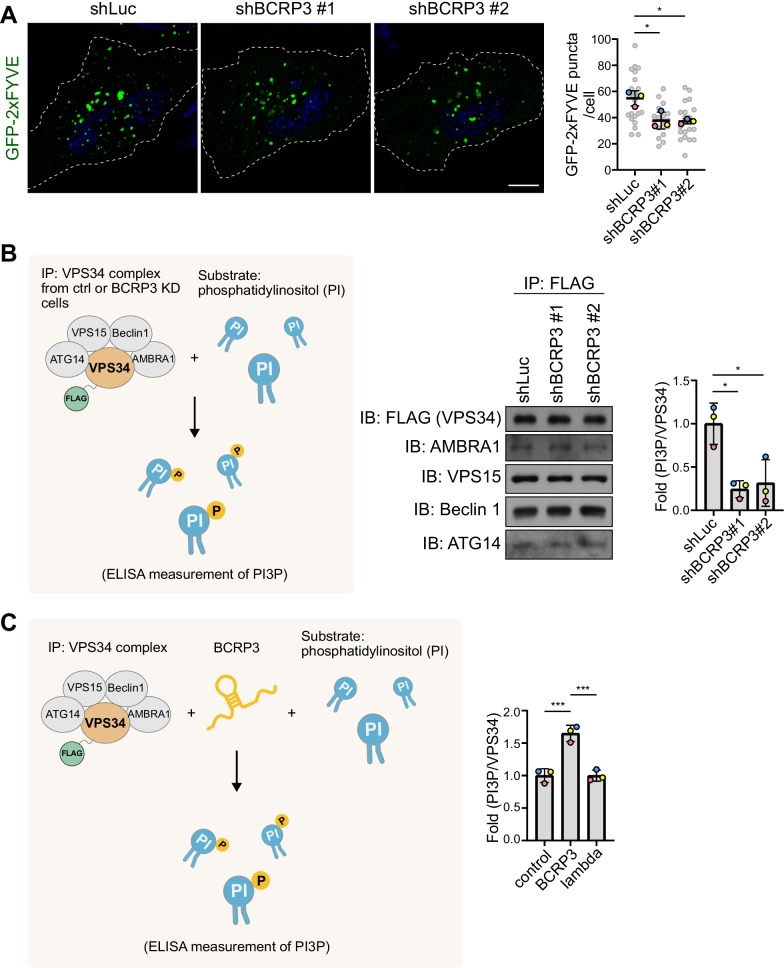
*BCRP3* stimulates the activity of VPS34 complex.** A** Confocal microscopy analysis of control or *BCRP3*-deficient HeLa cells, transiently transfected with GFP-2xFYVE and starved in EBSS for 1 h. Representative images are shown on the left and quantitative data are on the right. Bar, 10 μm. Data are means ± SD from three independent experiments and 7 cells per group per experiment were counted. *P* values are determined by one-way ANOVA with Tukey’s post hoc test, *P < 0.05.** B** FLAG-VPS34 was immunoprecipitated from control or *BCRP3*-deficient HeLa cells. The immunocomplexes were analyzed by Western blot (middle) or incubated with phosphatidylinositol (PI) substrate and ATP (left). PI3P production was measured by ELISA assay and normalized to FLAG-VPS34 protein levels (right). **C** FLAG-VPS34 immunoprecipitated from transfected HeLa cells was incubated with PI substrate and ATP, together with in vitro transcribed *BCRP3* or *lambda* RNA (left). PI3P production was measured by ELISA and normalized to FLAG-VPS34 protein levels (right). Data in (**B**) and (**C**) are means ± SD from three independent experiments. *P* values are determined by one-way ANOVA with Tukey’s post hoc test, *P < 0.05, ***P < 0.001

We next determined whether *BCRP3* could modulate the catalytic activity of VPS34 complex. Using the GFP-2xFYVE probe [38], we found that *BCRP3* knockdown in EBSS-cultured cells decreased the cellular level of PI3P, the product of VPS34 complex (Fig. 5A). Furthermore, VPS34 complex isolated from control cells showed a higher enzymatic activity in the in vitro kinase assay, compared to that isolated from *BCRP3* knockdown cells (Fig. 5B). To demonstrate a direct role of *BCRP3* in stimulating the enzymatic activity of VPS34 complex, we isolated VPS34 complex from FLAG-VPS34 transfected cells and incubated this complex with in vitro transcribed *BCRP3*. Addition of *BCRP3* significantly enhanced the enzymatic activity of VPS34 complex, whereas a control RNA failed to do so (Fig. 5C). These data provide compelling evidence for the stimulation of VPS34 enzymatic activity by *BCRP3*.

### *BCRP3* is upregulated by certain proteotoxic insults to contribute to aggrephagy-induction

**Fig. 6:**
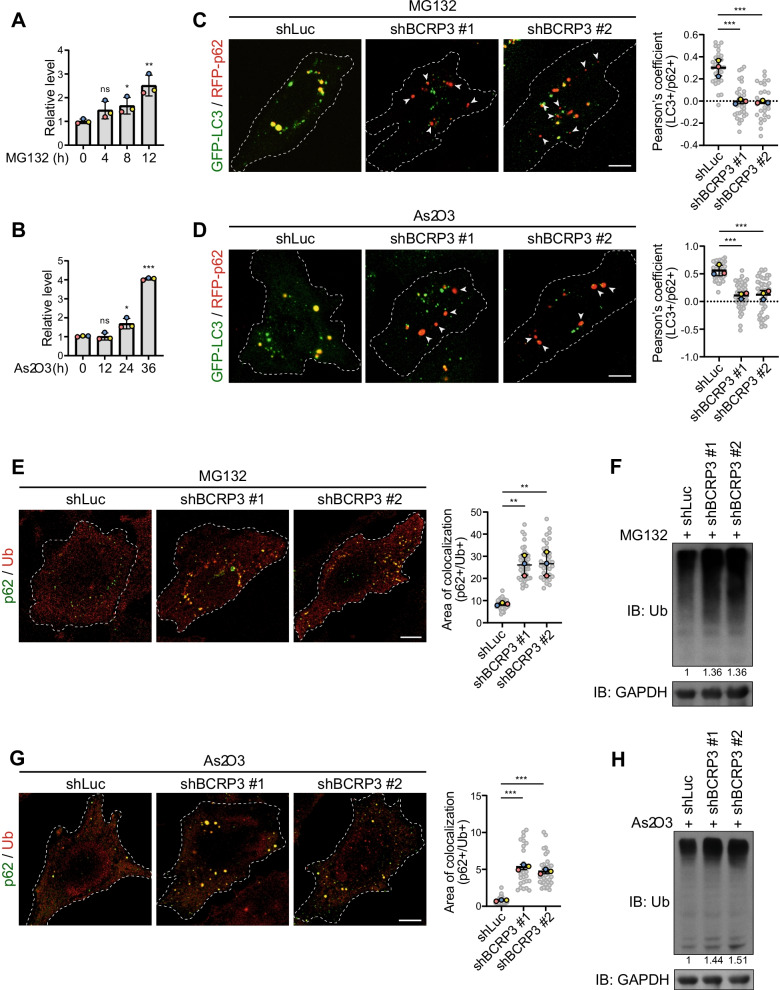
*BCRP3* is induced by certain proteotoxic stresses to enhance aggrephagy for protein quality control. **A, B** qRT-PCR analysis of *BCRP3* levels in HeLa cells treated with 10 µM MG132 (**A**) or 4 µM As_2_O_3_ (**B**) for the indicated time points. Data were normalized to *GAPDH* and expressed as means ± SD, n = 3. *P* values are determined by one-way ANOVA with Tukey’s post hoc test, *P < 0.05, **P < 0.01, ***P < 0.001; ns, not significant. **C, D** Control or *BCRP3*-deficient HeLa cells transfected with GFP-LC3 and RFP-p62 were treated with 10 µM MG132 for 8 h (**C**) or 4 µM As_2_O_3_ for 24 h (**D**) and analyzed by confocal microscopy (left). Arrowheads indicate the RFP-p62 puncta that are not colocalized with GFP-LC3. Bars, 10 μm. Pearson’s coefficients for the colocalization between GFP-LC3 and RFP-p62 signals were calculated by Image J and shown in the graphs (right). Data are means ± SD from three independent experiments and 10 cells per group per experiment were counted. *P* values are determined by one-way ANOVA with Tukey’s post hoc test, ***P < 0.001. **E**, **G** Control or *BCRP3*-deficient HeLa cells were treated with 10 µM MG132 for 8 h (**E**) or 4 µM As_2_O_3_ for 24 h (**G**), stained for p62 and ubiquitin, and analyzed by confocal microscopy (left). Bars, 10 μm. Area of colocalization between p62 and ubiquitin was calculated by Image J and shown in the graphs (right). Data are means ± SD from three independent experiments and 10 cells per group per experiment were counted. *P* values are determined by one-way ANOVA with Tukey’s post hoc test, **P < 0.01, ***P < 0.001. **F, H** Western blot analysis using control or *BCRP3*-deficient HeLa cells treated with 10 µM MG132 for 12 h (**F**) or 4 µM As_2_O_3_ for 24 h (**H**). Ubiquitinated protein levels were quantified and shown on the bottom

Having identified the function and mechanism of *BCRP3* in promoting autophagy, we next investigated whether *BCRP3* expression could be induced under certain autophagy-stimulating conditions. Our analysis found that *BCRP3* expression was upregulated in a time-dependent manner by treatment of cells with proteasome inhibitor MG132 or oxidative stressor As_2_O_3_ (Fig. 6A, B). In contrast, starvation and other proteotoxic stressors such as puromycin, HSP90 inhibitors 17-AAG and 17-DMAG did not affect *BCRP3* expression (Additional file 1, Fig. S6A). Of note, both MG132 and As_2_O_3_ are known to induce the formation of cytoplasmic protein aggregate called aggresome and aggresome-like induced structure (ALIS), respectively, and aggrephagy represent one mechanism for removing these aggregates [21, 39]. Thus, we investigated the effect of *BCRP3* on aggrephagy. Remarkably, *BCRP3* knockdown decreased aggrephagy in MG132 and As_2_O_3_-treated cells, as monitored by p62/LC3 double positive puncta (Fig. 6C, D). This was accompanied by increased accumulations of aggresome or ALIS (marked by Ub/p62 double positive puncta) and the total cellular ubiquitinated proteins (Fig. 6E–H). *BCRP3* knockdown in basal conditions, however, did not obviously increased the total cellular ubiquitinated proteins (Additional file 1, Fig. S6B). Thus, our study reveals the induction of *BCRP3* by proteotoxic stress to stimulate aggrephagy activity for protein quality control.

### Accumulation of growth/survival inhibitors and induction of TGF-β pathway by *BCRP3* deficiency in proteotoxicity

**Fig. 7:**
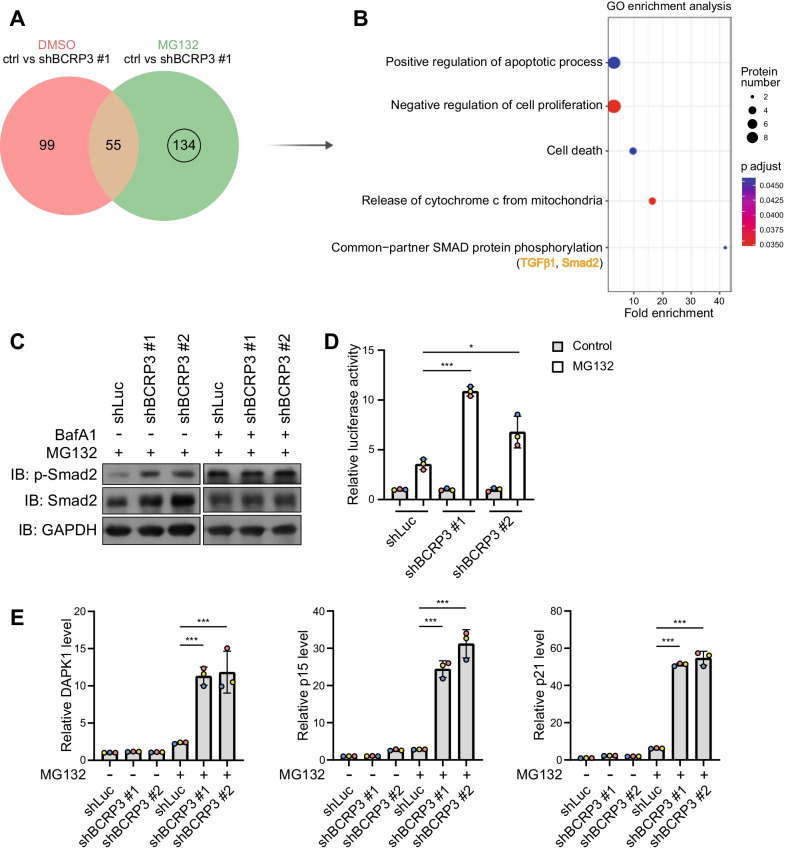
*BCRP3* deficiency in proteotoxicity leads to the accumulation of proteins involving in growth inhibition, cell death, and TGF-β/Smad2 signaling. **A** Venn diagram showing the numbers of enriched proteins after *BCRP3* knockdown together with or without 10 µM MG132 treatment for 12 h. **B** GO enrichment analysis of the 134 proteins shown in (**A)**. Selective enriched GO terms are shown by the order of fold enrichment (bottom to top). **C** Western blot analysis of indicated proteins in control or *BCRP3*-deficient HeLa cells treated with 10 µM MG132 together for 12 h together with or without 200 nM bafilomycin A1 for 2 h. **D** Control or *BCRP3*-deficient HeLa cells were transfected with 4 × SBE-Luc reporter construct, treated with 10 µM MG132 for 12 h and analyzed for luciferase activity. **E** qRT-PCR analysis of relative *DAPK1, p15,* and *p21* levels in control or *BCRP3*-deficient HeLa cells treated with 10 µM MG132 for 12 h. Data in (**D**), (**E**) are means ± SD from three independent experiments. *P* values are determined by one-way ANOVA with Tukey’s post hoc test, *P < 0.05, ***P < 0.001

Next, we sought to understand the impact of *BCRP3*-mediated aggrephagy on proteome landscape. We performed label-free quantitative LC–MS/MS analysis to detect the protein expression profiles in control and *BCRP3* knockdown cells treated with or without MG132. Proteins preferentially accumulated in MG132-treated *BCRP3* knockdown cells compared with MG132-treated control cells were defined with the following criteria: protein level [*BCRP3* knockdown/control] > 1.5, *P* < 0.05, and a total of 189 proteins were recovered. Among them, 55 were also accumulated in *BCRP3* knockdown cells under unstressed conditions and therefore were excluded (Fig. 7A). GO analysis of the remaining 134 proteins (listed in Additional file 1: Table S3) revealed “positive regulation of apoptotic process”, “negative regulation of cell proliferation”, “cell death”, “co-Smad protein phosphorylation and “release of cytochrome c from mitochondria” as the enriched GO terms (Fig. 7B). Among these GO terms, the enrichment of co-Smad protein phosphorylation is intriguing and both TGF-β1 and Smad2, a downstream effector of TGF-β signaling, appeared in this category (Fig. 7B). Of note, TGF-β pathway elicits potent cytostatic effects including growth arrest and apoptosis under certain cellular contexts [40]. We therefore evaluated the impact of *BCRP3* on TGF-β signaling in more details. Consistent with the data from proteomics analysis, *BCRP3* knockdown in MG132-treated cells increased Smad2 and p-Smad2 levels and these effects were abrogated by blocking the lysosome degradation with bafilomycin A1 (Fig. 7C), thus supporting a role of *BCRP3*-mediated aggrephagy in the selective degradation of Smad2. Furthermore, *BCRP3* knockdown in MG132-treated cells, but not control cells, increased the activity of a Smad-responsive reporter 4xSBE-Luc and the mRNA levels of *DAPK1*, *p15INK4B* (also known as *CDKN2B*) and *p21Cip1* (also known as *CDKN1A*) (Fig. 7D, E), which are the cytostatic effectors of TGF-β/Smad2 pathway [41–43]. These findings identify the function of *BCRP3* in selective downregulation of TGF-β signaling, cell death-promoting factors and proliferation inhibitors during proteotoxicity-induced aggrephagy.

### *BCRP3* prevents proteotoxicity-induced growth arrest and apoptosis partly through downregulating TGF-β signaling

**Fig. 8:**
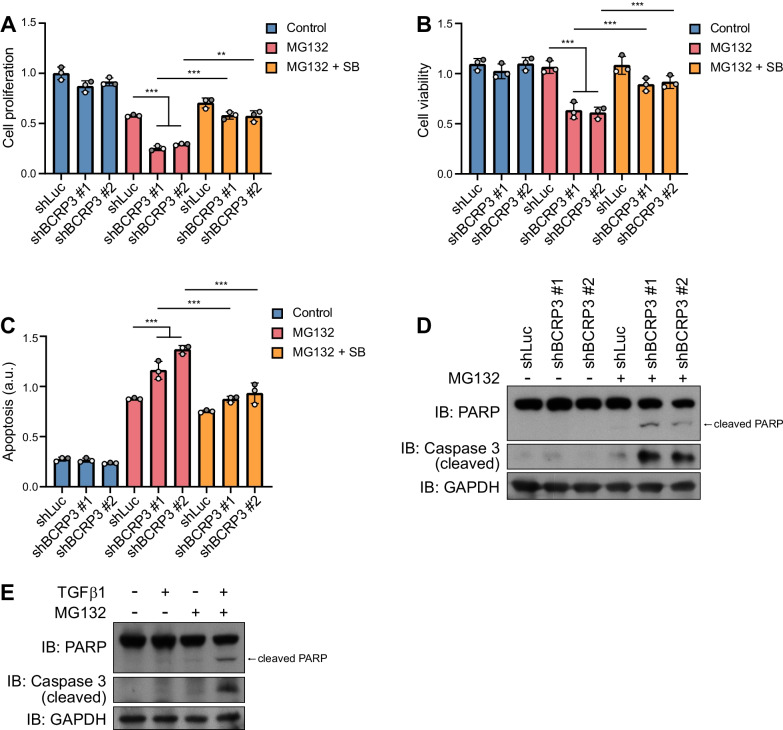
*BCRP3* promotes proliferation and survival in proteotoxic cells. **A** BrdU incorporation assay for control or *BCRP3*-deficient HeLa cells treated with 10 µM MG132, 4 µM SB431542 and incubated with BrdU for 24 h. **B, C** MTT (**B**), and apoptosis (**C**) assays for control or *BCRP3*-deficient HeLa cells treated with 10 µM MG132 for 12 h followed by 4 µM SB431542 for 2 h. Data in (**A**), (**B**), and (**C**) are means ± SD from three independent experiments. *P* values are determined by two-way ANOVA with Tukey’s post hoc test, **P < 0.01, ***P < 0.001. **D, E** Western blot analysis of indicated proteins in HeLa cells treated with or without 10 µM MG132 for 16 h, followed by 5 ng/ml TGF-β for 2 h

The identification of *BCRP3* functions in aggrephagy and proteome landscape alteration prompted us to investigate its impact on cell proliferation and survival under proteotoxicity. Through BrdU incorporation and MTT assays, we found that *BCRP3* knockdown decreased proliferation and viability, respectively, in MG132-treated cells but not control cells (Fig. 8A, B). *BCRP3* knockdown also enhanced apoptosis, PARP cleavage and caspase 3 cleavage in MG132-treated cells but not untreated cells (Fig. 8C, D). Remarkably, the effects of *BCRP3* knockdown on cell proliferation, viability and apoptosis were all reversed partially by blocking TGF-β signaling with the TβRI inhibitor SB431542 (Fig. 8A-C). Furthermore, enforced activation of TGF-β pathway in MG132-treated cells enhanced apoptosis (Fig. 8E). Together, our study uncovers a role of *BCRP3* in maintaining cell proliferation and viability and preventing apoptosis during proteotoxicity, which are mediated in part through downregulating TGF-β signaling.

## Discussion

Transcriptional, post-transcriptional and posttranslational regulations are the three major mechanisms governing many cellular processes, including autophagy. Besides these commonly employed regulatory mechanisms, a novel “riboregulation” concept has recently emerged, which is referred to as a regulatory mechanism with the following features [44]. First, the direct interaction between a regulatory RNA and its target protein can lead to a functional change of the target protein. Furthermore, the level or activity of the regulatory RNA and/or target protein can be altered by a biological cue. In this study, we identify lncRNA *BCRP3* as a positive regulator of autophagy by binding and activating the VPS34 complex, a core component in both bulk and selective autophagy processes. Furthermore, *BCRP3* is upregulated under certain proteotoxic conditions to enhance aggrephagy activity for the clearance of ubiquitinated protein aggregates. These properties of *BCRP3* indicate its role as a novel “riboregulator” of autophagy. To date, only couple riboregulatory mechanisms of autophagy have been identified, including lncRNA *NBR2*-mediated AMPK activation in controlling energy stress-induced autophagy [26] and small ncRNA *Vault RNA1-1*-modulated p62 oligomerization in suppressing aggrephagy [45]. Notably, a recent high-throughput screen identified 63 lncRNAs that affect autophagosome numbers in basal or Torin 1-treated conditions [46], supporting a profound role of lncRNAs in autophagy regulation. However, whether any of these lncRNAs fulfills the riboregulation characters remains to be studied.

We show that *BCRP3* functions as a RNA activator of the VPS34 complex. Importantly, *BCRP3* affects neither the cellular abundance nor the integrity of VPS34 complex. Furthermore, *BCRP3* is able to stimulate the activity of purified VPS34 complex in vitro, without the need of a membrane environment. These findings suggest that *BCRP3* binding to the VPS34 complex results in a conformational change to elevate its enzymatic activity, even though we cannot rule out the possibility for an additional role of *BCRP3* in promoting the membrane targeting of VPS34 complex. Of note, numerous post-translational mechanisms have been identified to regulate the activity of VPS34 complex [7, 47], highlighting its high susceptibility to conformational change-induced regulation. In addition, the activity of VPS34 can also be modulated by various binding partners. For instance, recent structural analyses revealed that the tight association of VPS15 with VPS34 restrains the activation loop of VPS34 to inhibit its catalytic activity [48], and the recruitment of NRBF2 to VPS34 complex facilitates an allosteric unleashing of the VPS34 kinase domain to drive VPS34 activation [49]. Since the detailed binding footprints of *BCRP3* on VPS34 complex are currently unknown, the exact mechanism for its stimulatory function in VPS34 activity awaits further study.

Intriguingly, *BCRP3* expression is upregulated by proteasome inhibitor MG132 and oxidative stressor arsenic trioxide, both of which are known to increase cellular misfolded proteins and ubiquitinated protein aggregates. However, several other agents that trigger proteotoxic stress, such as puromycin and HSP90 inhibitors, do not alter *BCRP3* level. Although the mechanism for *BCRP3* induction by the particular proteotoxic stressors remain unclear, we showed that this elevated *BCRP3* expression contributes to aggrephagy induction for an efficient removal of misfolded protein aggregates, consistent with a crucial role of VPS34 complex in this type of selective autophagy [19, 21]. Furthermore, although it is well documented that aggrephagy is induced upon proteasome impairment to compensate the degradative capacity [50–52], the exact molecular mechanisms for this induction have not been completely understood. Our study identifies *BCRP3* as a new player that mediates the cross-talk between ubiquitin–proteasome system and autophagy process for protein quality control.

Although the detailed molecular mechanisms of aggrephagy have been dissected [53, 54], the impacts of aggrephagy on proteome landscapes and cell signaling are not well understood. Our study uncovered that *BCRP3* depletion under proteotoxicity leads to a preferential accumulation of proteins involving in the growth inhibition, cell death, apoptosis, and TGF-β/Smad2 pathway. Consistent with this proteome alterations, *BCRP3* deficiency in proteotoxic cells compromises cell viability and proliferation and increases apoptosis and these effects are mediated in part through the activation of TGF-β/Smad2 pathway. Thus, our findings highlight the importance of *BCRP3*-mediated aggrephagy in the selective removal of a set of cell death and growth inhibitory proteins to maintain cell fitness during proteotoxicity. Notably, even though proteins accumulated in the proteotoxic *BCRP3* deficient cells could be direct cargos of aggrephagy or indirectly affected by aggrephagy cargos, at least some of these proteins are degraded through a *BCRP3*-dependent and lysosome-dependent manner during proteotoxicity, implying that they are direct cargos of *BCRP3*-mediated aggrephagy. Identification of the specific set of aggrephagy cargos regulated by *BCRP3* would require further systematic analyses, such as LC3- and cargo receptor-based proximity labeling [55]. Finally, since TGF-β pathway blockage does not completely reverse *BCRP3* deficiency-induced detrimental effects in proteotoxic cells, other accumulated proteins, including proteins with anti-proliferation/pro-apoptosis functions or toxic protein aggregates, likely contribute to the cell growth and survival defects.

*BCRP3* was identified based on its downregulation in many types of tumor tissues, compared with the normal tissues. However, whether *BCRP3* plays a suppressive role in tumor initiation through its autophagy-enhancing function remains unclear. Studying the tumor-initiation function often requires the utilization of genetically modified mouse models, but the lack of murine homologue of *BCRP3* precludes such study. Although the function of *BCRP3* in cancer remains elusive, our finding for the upregulation of *BCRP3* in response to certain proteotoxic stresses and its effect on aggrephagy induction for maintaining proteostasis and cell viability imply an impact of *BCRP3* on preventing pathological states associated with the accumulation of proteinaceous inclusions. These proteinopathies represent a large group of disease states, including various neurodegenerative diseases, and evidence has emerged that autophagy plays protective roles against these disease states [56, 57]. Future study will aim to interrogate the function and expression of *BCRP3* in these human pathological states.

## Conclusions

We identified lncRNA *BCRP3* as a riboactivator of autophagy through its binding and stimulating the catalytic activity of VPS34 complex. In response to certain proteotoxic stresses, *BCRP3* is upregulated to enhance aggrephagy activity. *BCRP3* deficiency under proteotoxicity not only compromises protein quality control but leads to the accumulation of anti-proliferation and cell death proteins/signaling molecules to suppress cell proliferation and survival.

## Supplementary Information

**Additional file 1: Fig. S1. BCRP3** expression in various human tissues. RNA-seq data from indicated tissues were retrieved from GTEx database. **Fig. S2.** *BCRP3* desensitizes cancer cells to chemotherapeutic agent without affecting DNA damage sensing/repair. **A** HCT116 cells stably expressing vector or *BCRP3* were treated with or without 5 μM 5-FU for 48 h, and cell viability was determined by MTT assay. The expression levels of *BCRP3* were analyzed by qRT-PCR and shown on the right. Data are means ± SD from three independent experiments. *P* values are determined by unpaired t-test, ***P < 0.001; ns, not significant. **B** Western blot analysis of indicated proteins from cells as in **A** and treated with 5 μM 5-FU for indicated time points. **C **HCT116 cells stably expressing *BCRP3* were treated with 5 μM 5-FU for 48 h, followed by 200 nM bafilomycin A1 or 10 mM 3-MA for 2 h. Cell viability was determined by MTT assay. Data are means ± SD from three independent experiments. *P* values are determined by one-way ANOVA with Tukey’s post hoc test, **P < 0.01, ***P < 0.001. **Figure S3.** *BCRP3* promotes autophagosome formation. **A** Immunofluorescence staining of LC3 in HCT116 cells stably expressing vector or *BCRP3* and starved in EBSS for 2 h. Representative confocal images are shown on the left and quantitative data are on the right. Bar, 10 μm. **B** Immunofluorescence staining of LC3 in 293T cells stably expressing control or *BCRP3* shRNAs and treated with or without 200 nM bafilomycin A1 for 2 h. Representative confocal images are shown on the left and quantitative data are on the middle. Bar, 10 μm. The expression levels of *BCRP3* were analyzed by qRT-PCR and shown on the right. Data in (**A**), (**B**) are means ± SD from three independent experiments and 30 cells per group per experiment were counted. *P* values are determined by unpaired t-test (**A**) or one-way ANOVA with Tukey’s post hoc test (**B**), **P < 0.01, ***P < 0.001. **C **Western blot analysis of LC3 in *BCRP3*-deficient 293T cells treated with or without 200 nM bafilomycin A1 for 2 h. **Figure S4.** *BCRP3 *deficiency decreases WIPI2 puncta. Immunofluorescence staining of WIPI2 in control or *BCRP3*-deficient HeLa cells starved in EBSS for 2 h. Representative confocal images are shown on the left and quantitative data are on the right. Bar, 10 μm. Arrowheads indicate the WIPI2 puncta. Data are means ± SD from three independent experiments and 10 cells per group per experiment were counted. *P* values are determined by one-way ANOVA with Tukey’s post hoc test, *P < 0.05. **Figure S5.** *BCRP3* deficiency does not affect VPS34 complex abundance and integrity and Beclin 1 oligomerization. **A** Subunit composition of VPS34 complex in control or *BCRP3*-deficient HeLa cells. VPS34 complexes were immunoprecipitated and the immunocomplexes were analyzed by Western blot with indicated antibodies. **B** Oligomerization of Beclin 1 in *BCRP3*-deficient HeLa cells. Lysates from control or *BCRP3*-deficient HeLa cells co-transfected with HA-/FLAG-Beclin 1 were immunoprecipitated with anti-FLAG M2 beads. The interaction between HA- and FLAG-tagged Beclin 1 was analyzed by Western blot with indicated antibodies. **Figure S6.** *BCRP3* expression and effect under different conditions. **A** *BCRP3* expression in responses to different stresses. qRT-PCR analysis of *BCRP3* levels in HeLa cells starved in EBSS, or treated with 10 μg/ml puromycin, 10 μM 17-AAG, or 10 μM 17-DMAG for the indicated time points. Data are means ± SD from three independent experiments. *P* values are determined by one-way ANOVA with Tukey’s post hoc test; ns, not significant. **B** Western blot analysis using control or *BCRP3*-deficient HeLa cells cultured in normal conditions. Ubiquitinated protein levels were quantified and shown on the bottom. **Table S1.** Antibody details. **Table S2.** Sequences of PCR primers. **Table S3.** List of proteins accumulated in *BCRP3*-deficient cells under proteotoxicity.

## Data Availability

The original mass spectrometry data for proteome analysis are deposited to the ProteomeXchange Consortium via PRIDE partner repository with the project accession number PXD030756. All other data supporting the findings of this study are available from the corresponding author upon reasonable request.

## Abbreviations

3-MA: 3-Methyladenine
5-FU: 5-Fluorouracil
ALIS: Aggresome-like induced structure
*BCRP3*: Break point region pseudogene 3
EBSS: Earle's Balanced Salt Solution
FBS: Fetal bovine serum
FDR: False discovery rate
FISH: Fluorescence in situ hybridization
GO: Gene ontology
LC–MS/MS: Liquid chromatography-tandem mass spectrometry
LIR: LC3-interacting region
lncRNAs: Long non-coding RNAs
MEM: Minimum essential medium
NCBI: National Center for Biotechnology Information
PI3K: Phosphatidylinositol-3-kinase
PI3P: Phosphatidylinositol 3-phosphate
PS: Penicillin/streptomycin
qRT-PCR: Quantitative real-time PCR
RPMI: Roswell Park Memorial Institute
TβRI: Type I TGF-β receptor
TCGA: The Cancer Genome Atlas
