# Correction to: Long noncoding RNA BCRP3 stimulates VPS34 and autophagy activities to promote protein homeostasis and cell survival

**DOI:** 10.1186/s12929-022-00842-x

**Published:** 2022-08-14

**Authors:** Ruei Liang Yan, Chiu Lin Luan, Chun Chieh Liao, Li Heng Liu, Fei Yun Chen, Hsin Yi Chen, Ruey Hwa Chen

**Affiliations:** 1 Institute of Biological Chemistry, Academia Sinica, Taipei, 115 Taiwan; 2 Institute of Molecular Medicine, College of Medicine, National Taiwan University, Taipei, 100 Taiwan; 3 Genome and Systems Biology Degree Program, College of Life Science, National Taiwan University, Taipei, 106 Taiwan; 4 Graduate Institute of Cancer Biology and Drug Discovery, College of Medical Science and Technology, Taipei Medical University, Taipei, 110 Taiwan; 5 Ph.D. Program for Cancer Molecular Biology and Drug Discovery, College of Medical Science and Technology, Taipei Medical University, Taipei, 110 Taiwan

## Correction: Journal of Biomedical Science (2022) 29:30 https://doi.org/10.1186/s12929-022-00815-0

After the publication of this article [1], we noted that the GAPDH blot of the Fig. 7C right panel is incorrect. The corrected Fig. 7C is included below.

**Fig. 7:**
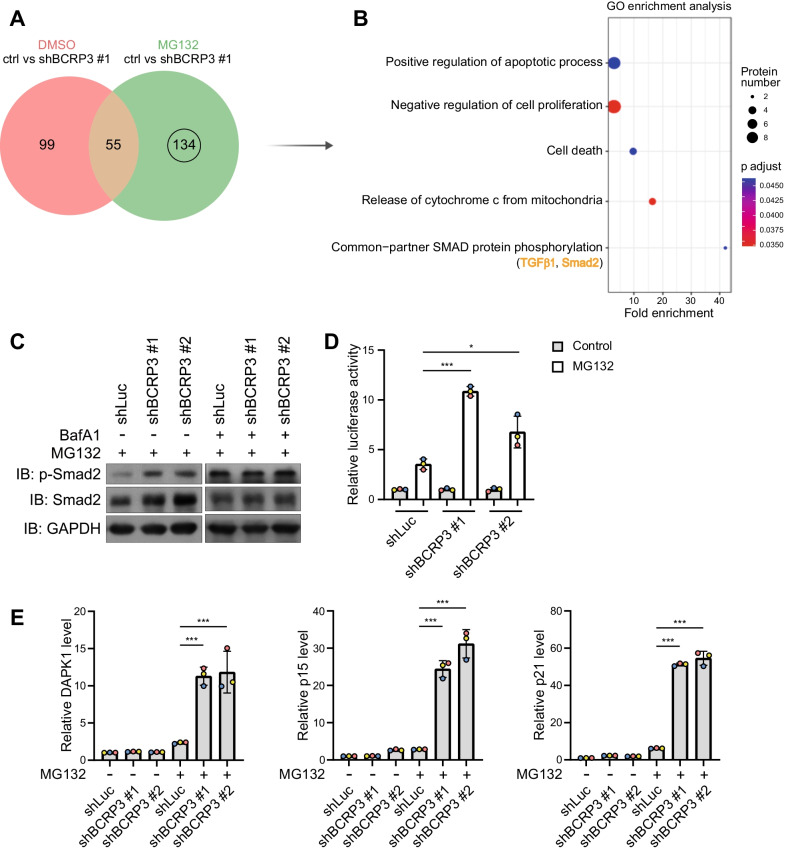
BCRP3 defciency in proteotoxicity leads to the accumulation of proteins involving in growth inhibition, cell death, and TGF-β/Smad2 signaling. **A** Venn diagram showing the numbers of enriched proteins after BCRP3 knockdown together with or without 10 µM MG132 treatment for 12 h. **B** GO enrichment analysis of the 134 proteins shown in (**A**). Selective enriched GO terms are shown by the order of fold enrichment (bottom to top). **C** Western blot analysis of indicated proteins in control or BCRP3-defcient HeLa cells treated with 10 µM MG132 together for 12 h together with or without 200 nM baflomycin A1 for 2 h. **D** Control or BCRP3-defcient HeLa cells were transfected with 4 × SBE-Luc reporter construct, treated with 10 µM MG132 for 12 h and analyzed for luciferase activity. **E** qRT-PCR analysis of relative DAPK1, p15, and p21 levels in control or BCRP3-defcient HeLa cells treated with 10 µM MG132 for 12 h. Data in (**D**), (**E**) are means ± SD from three independent experiments. P values are determined by one-way ANOVA with Tukey’s post hoc test, *P < 0.001
