# Supplementary material for: Long noncoding RNA BCRP3 stimulates VPS34 and autophagy activities to promote protein homeostasis and cell survival
Source: J Biomed Sci. 2022 May 10;29:30. doi: 10.1186/s12929-022-00815-0 (PMC9087997; doi:10.1186/s12929-022-00815-0)
Supplement: Supplementary file 1 — Additional file 1: Fig. S1. BCRP3 expression in various human tissues. RNA-seq data from indicated tissues were retrieved from GTEx database. Fig. S2. BCRP3 desensitizes cancer cells to chemotherapeutic agent without affecting DNA damage sensing/repair. A HCT116 cells stably expressing vector or BCRP3 were treated with or without 5 μM 5-FU for 48 h, and cell viability was determined by MTT assay. The expression levels of BCRP3 were analyzed by qRT-PCR and shown on the right. Data are means ± SD from three independent experiments. P values are determined by unpaired t-test, ***P < 0.001; ns, not significant. B Western blot analysis of indicated proteins from cells as in A and treated with 5 μM 5-FU for indicated time points. C HCT116 cells stably expressing BCRP3 were treated with 5 μM 5-FU for 48 h, followed by 200 nM bafilomycin A1 or 10 mM 3-MA for 2 h. Cell viability was determined by MTT assay. Data are means ± SD from three independent experiments. P values are determined by one-way ANOVA with Tukey’s post hoc test, **P < 0.01, ***P < 0.001. Figure S3. BCRP3 promotes autophagosome formation. A Immunofluorescence staining of LC3 in HCT116 cells stably expressing vector or BCRP3 and starved in EBSS for 2 h. Representative confocal images are shown on the left and quantitative data are on the right. Bar, 10 μm. B Immunofluorescence staining of LC3 in 293T cells stably expressing control or BCRP3 shRNAs and treated with or without 200 nM bafilomycin A1 for 2 h. Representative confocal images are shown on the left and quantitative data are on the middle. Bar, 10 μm. The expression levels of BCRP3 were analyzed by qRT-PCR and shown on the right. Data in (A), (B) are means ± SD from three independent experiments and 30 cells per group per experiment were counted. P values are determined by unpaired t-test (A) or one-way ANOVA with Tukey’s post hoc test (B), **P < 0.01, ***P < 0.001. C Western blot analysis of LC3 in BCRP3-deficient 293T cells treated with or without 2 [file 12929_2022_815_MOESM1_ESM.docx]

**Additional File 1**

**Long noncoding RNA *BCRP3* stimulates VPS34 and autophagy activities to promote protein homeostasis and cell survival**

**Additional File 1**

**Additional Figure 1-6**

**Additional Table 1-3**

**
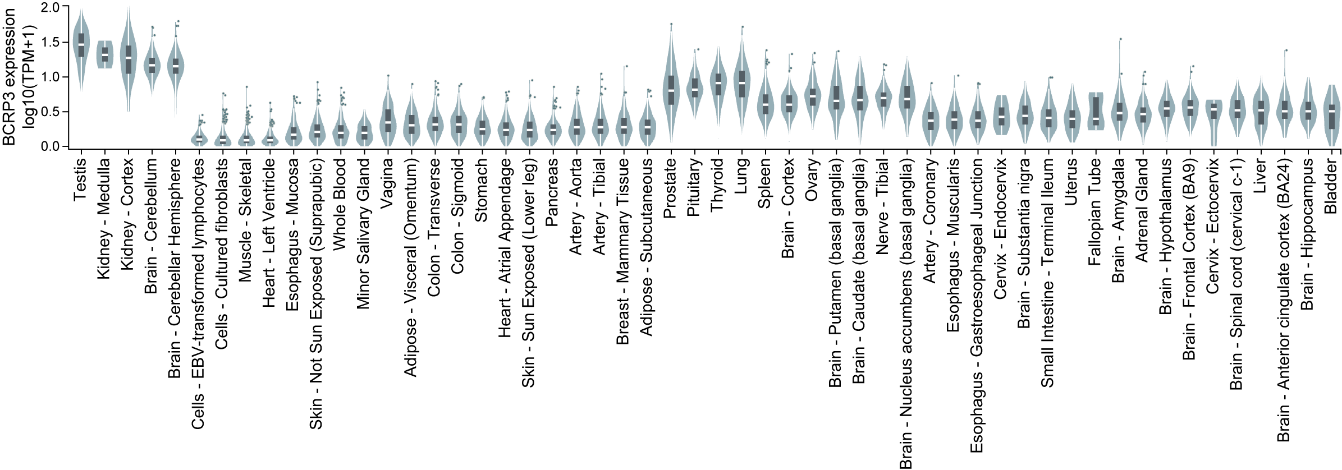
**

**Fig. S1** *BCRP3* expression in various human tissues. RNA-seq data from indicated tissues were retrieved from GTEx database.

**
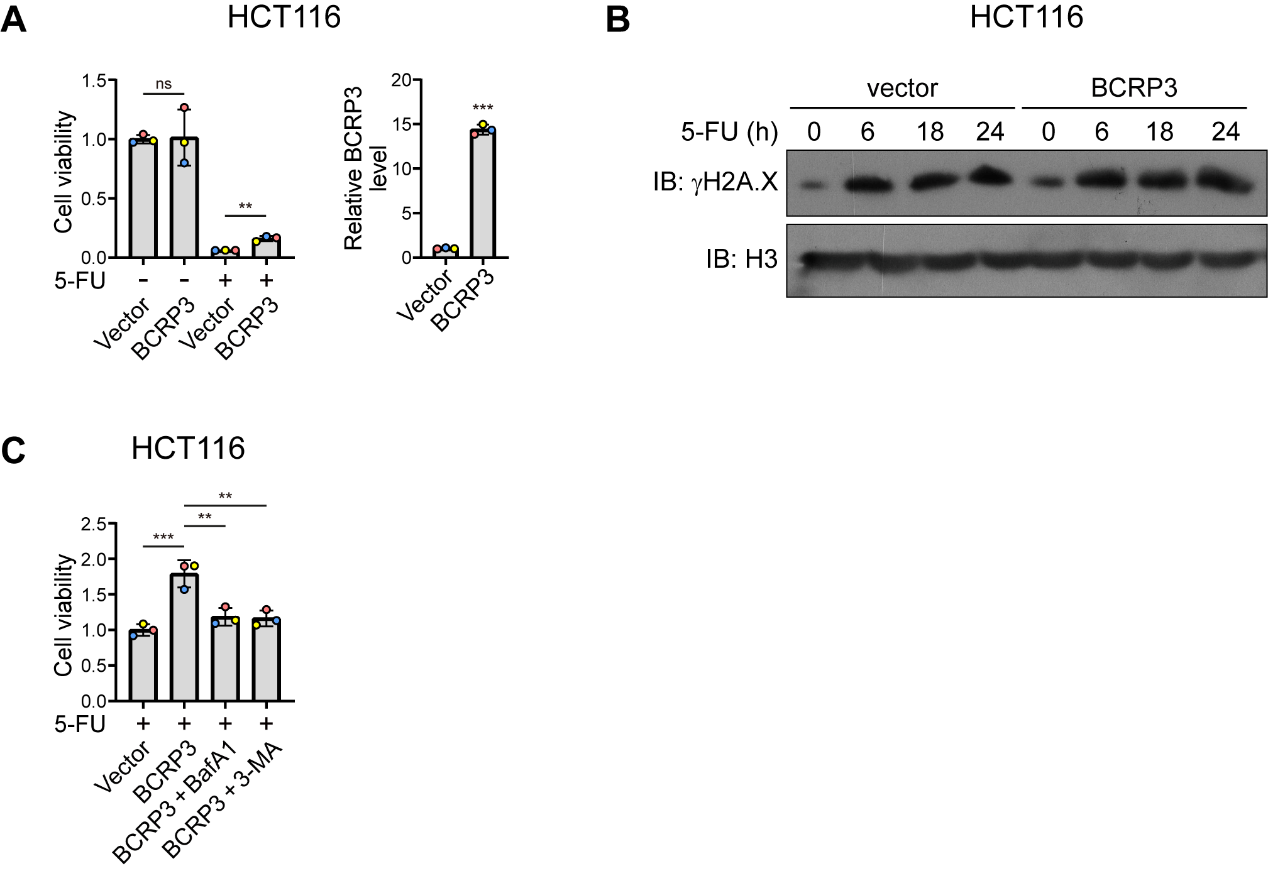
**

**Fig. S2** *BCRP3* desensitizes cancer cells to chemotherapeutic agent without affecting DNA damage sensing/repair. **A** HCT116 cells stably expressing vector or *BCRP3* were treated with or without 5 μM 5-FU for 48 hr, and cell viability was determined by MTT assay. The expression levels of *BCRP3* were analyzed by qRT-PCR and shown on the right. Data are means ± SD from three independent experiments. *P* values are determined by unpaired t-test, ****P* < 0.001; ns, not significant. **B** Western blot analysis of indicated proteins from cells as in **A** and treated with 5 μM 5-FU for indicated time points. **C** HCT116 cells stably expressing *BCRP3* were treated with 5 μM 5-FU for 48 hr, followed by 200 nM bafilomycin A1 or 10 mM 3-MA for 2 hr. Cell viability was determined by MTT assay. Data are means ± SD from three independent experiments. *P* values are determined by one-way ANOVA with Tukey’s post hoc test, ***P* < 0.01, ****P* < 0.001.

**
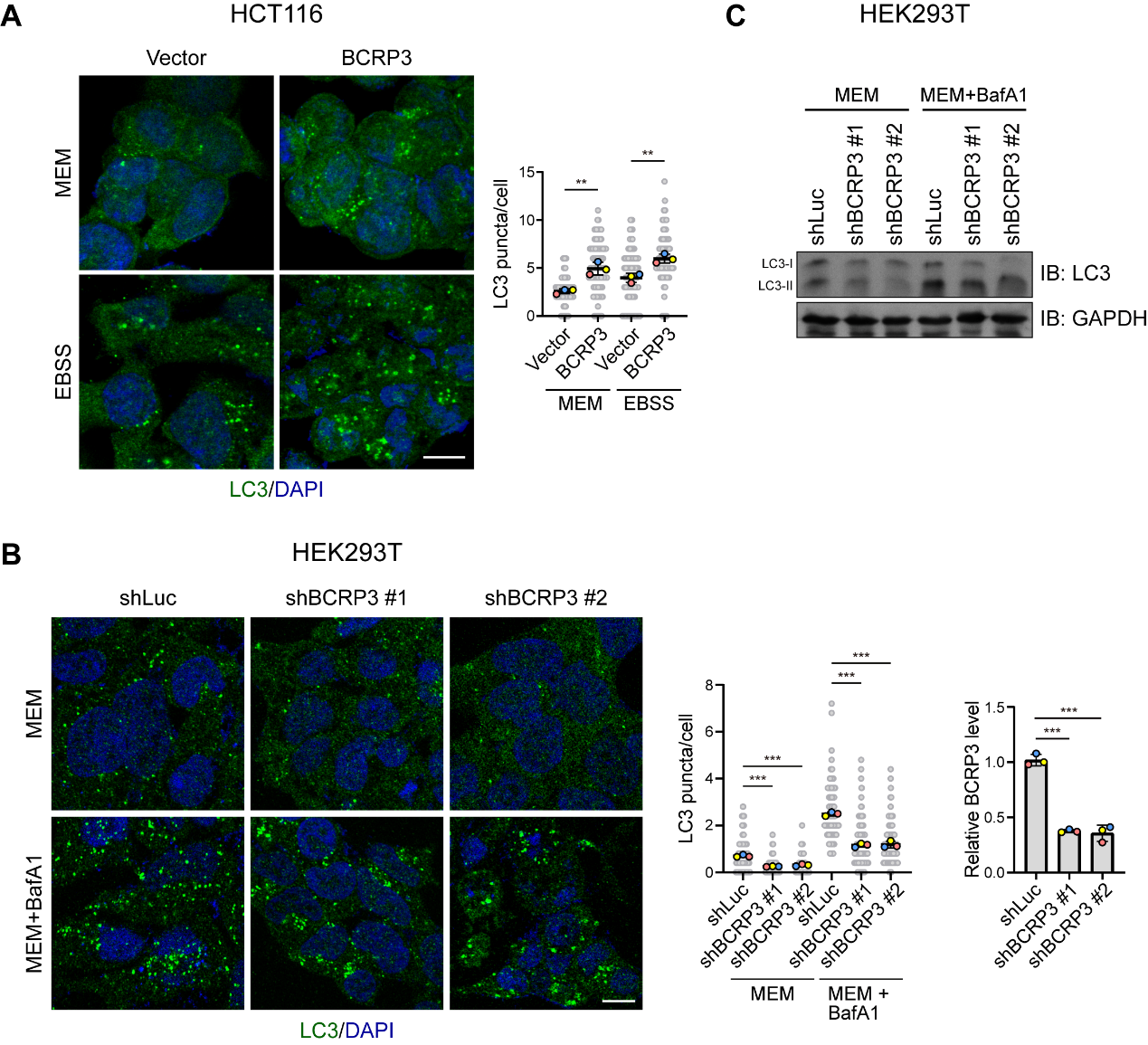
**

**Figure S3.** *BCRP3* promotes autophagosome formation. **A** Immunofluorescence staining of LC3 in HCT116 cells stably expressing vector or *BCRP3* and starved in EBSS for 2 hr. Representative confocal images are shown on the left and quantitative data are on the right. Bar, 10 μm. **B** Immunofluorescence staining of LC3 in 293T cells stably expressing control or *BCRP3* shRNAs and treated with or without 200 nM bafilomycin A1 for 2 hr. Representative confocal images are shown on the left and quantitative data are on the middle. Bar, 10 μm. The expression levels of *BCRP3* were analyzed by qRT-PCR and shown on the right. Data in (**A**), (**B**) are means ± SD from three independent experiments and 30 cells per group per experiment were counted. *P* values are determined by unpaired t-test (**A**) or one-way ANOVA with Tukey’s post hoc test (**B**), ***P* < 0.01, ****P* < 0.001. **C** Western blot analysis of LC3 in *BCRP3*-deficient 293T cells treated with or without 200 nM bafilomycin A1 for 2 hr.

**
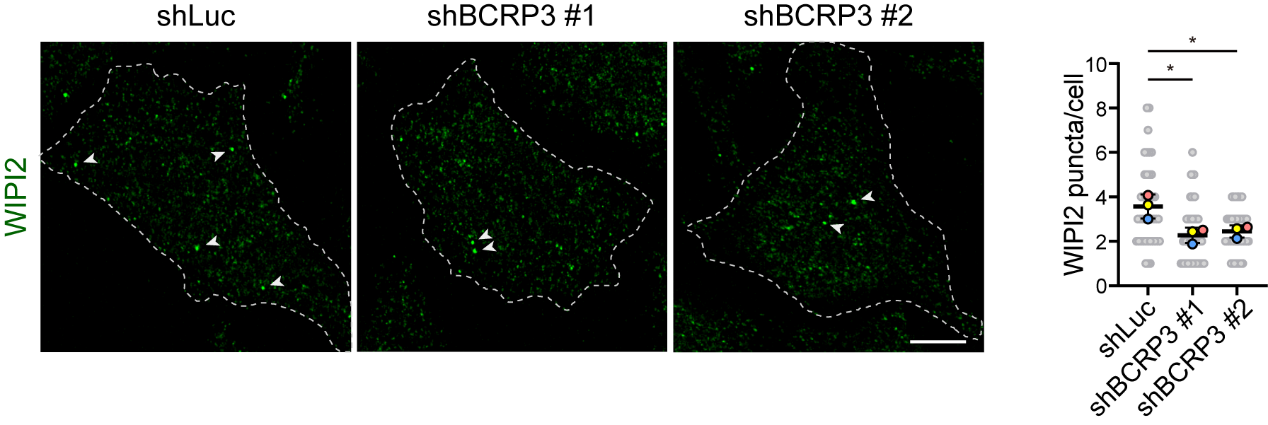
**

**Figure S4.** *BCRP3* deficiency decreases WIPI2 puncta. Immunofluorescence staining of WIPI2 in control or *BCRP3*-deficient HeLa cells starved in EBSS for 2 hr. Representative confocal images are shown on the left and quantitative data are on the right. Bar, 10 μm. Arrowheads indicate the WIPI2 puncta. Data are means ± SD from three independent experiments and 10 cells per group per experiment were counted. *P* values are determined by one-way ANOVA with Tukey’s post hoc test, **P* < 0.05.

**
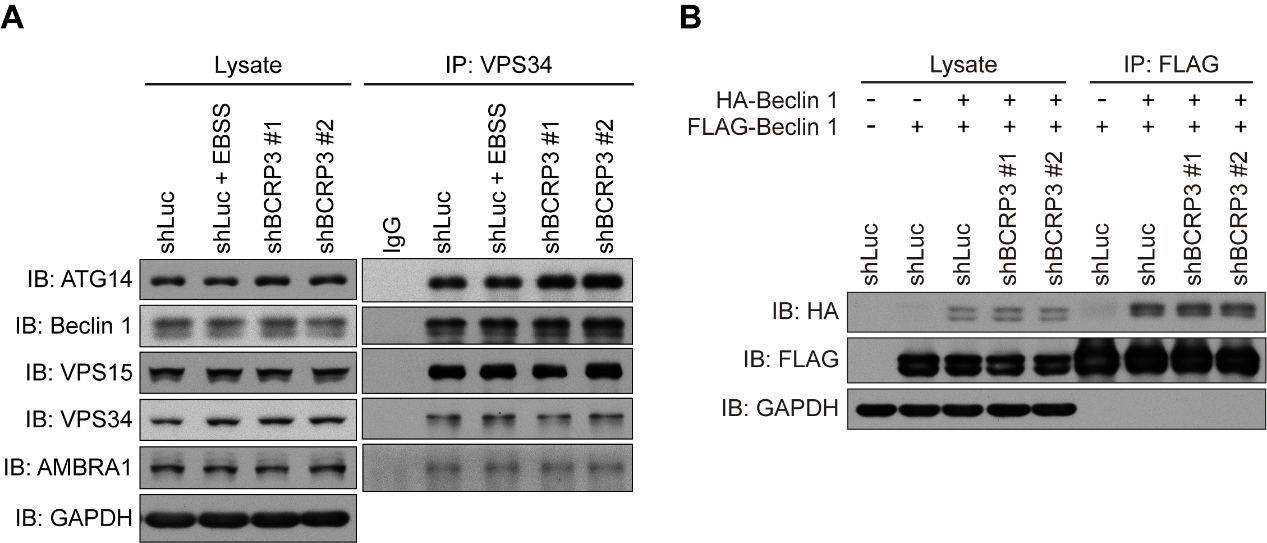
**

**Figure S5.** *BCRP3* deficiency does not affect VPS34 complex abundance and integrity and Beclin 1 oligomerization. **A** Subunit composition of VPS34 complex in control or *BCRP3*-deficient HeLa cells. VPS34 complexes were immunoprecipitated and the immunocomplexes were analyzed by Western blot with indicated antibodies. **B** Oligomerization of Beclin 1 in *BCRP3*-deficient HeLa cells. Lysates from control or *BCRP3*-deficient HeLa cells co-transfected with HA-/FLAG-Beclin 1 were immunoprecipitated with anti-FLAG M2 beads. The interaction between HA- and FLAG-tagged Beclin 1 was analyzed by Western blot with indicated antibodies.

**
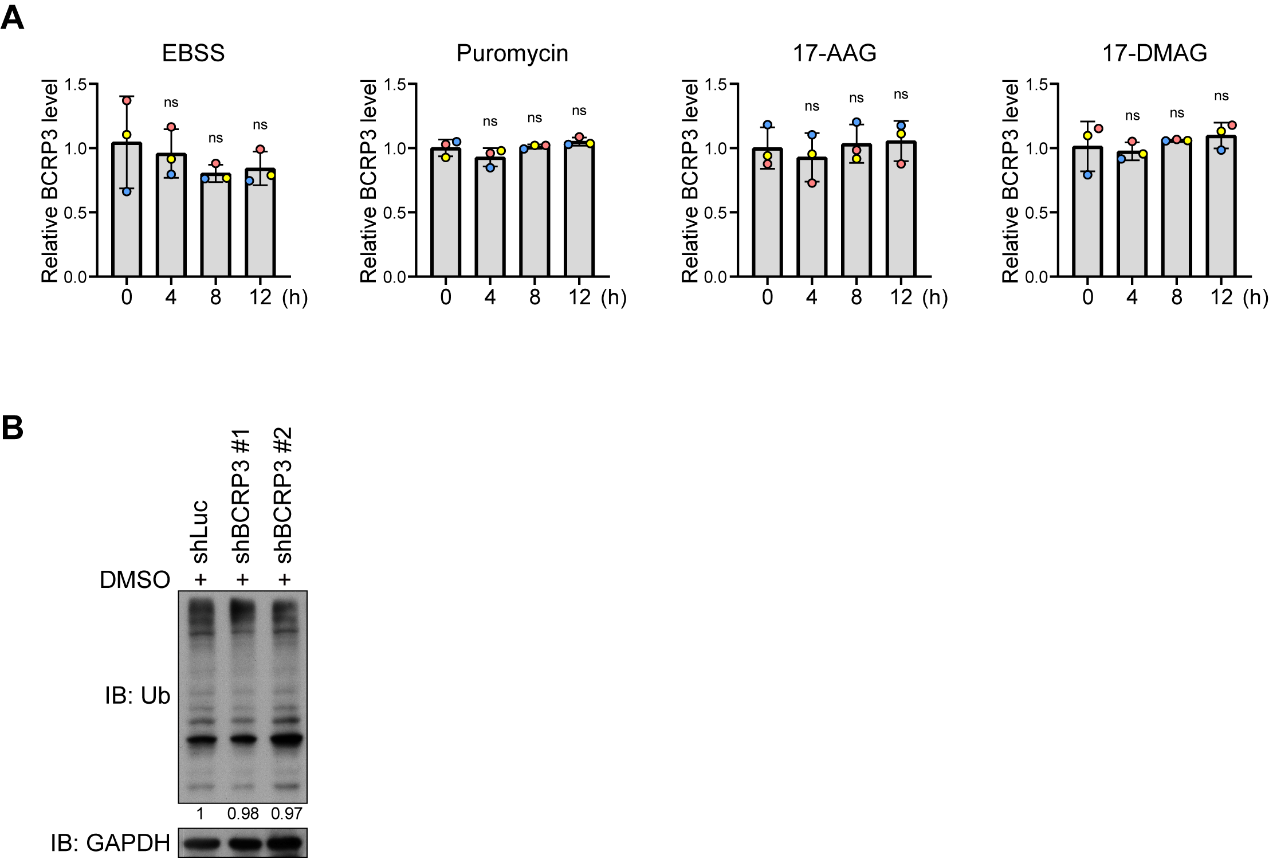
**

**Figure S6.** *BCRP3* expression and effect under different conditions. **A** *BCRP3* expression in responses to different stresses. qRT-PCR analysis of *BCRP3* levels in HeLa cells starved in EBSS, or treated with 10 μg/ml puromycin, 10 μM 17-AAG, or 10 μM 17-DMAG for the indicated time points. Data are means ± SD from three independent experiments. *P* values are determined by one-way ANOVA with Tukey’s post hoc test; ns, not significant. **B** Western blot analysis using control or *BCRP3*-deficient HeLa cells cultured in normal conditions. Ubiquitinated protein levels were quantified and shown on the bottom.

| **Table S1: Antibody details** | | | | |
| --- | --- | --- | --- | --- |
| **Protein** | **Company** | **Cat. number** | **Source** | **Titer** |
| LC3 | Cell Signaling | 2775 | Rabbit | WB (1:1000) |
| LC3 | Abcam | ab48394 | Rabbit | IF (1:200) |
| GAPDH | GeneTex | GTX100118 | Rabbit | WB (1:5000) |
| Atg16L1 | MBL | PM040 | Rabbit | WB (1:1000) |
| WIPI2 | Abcam | ab105459 | Rabbit | IF (1:200) |
| ULK1 | Santa Cruz | sc-33182 | Rabbit | WB (1:1000) |
| ULK1-pS757 | Cell Signaling | 14202 | Rabbit | WB (1:1000) |
| ULK1-pS317 | Cell Signaling | 6887 | Rabbit | WB (1:1000) |
| Atg13 | Sigma-Aldrich | SAB4200100 | Rabbit | WB (1:1000) |
| Atg13-pS318 | Rockland | 600-401-C49 | Rabbit | WB (1:1000) |
| VPS34 | Cell Signaling | 4263 | Rabbit | WB (1:1000) |
| VPS34 | Echelon Biosciences | Z-R015 | Rabbit | IP (1:150) |
| Beclin 1 | Novus | NB110-87318 | Rabbit | WB (1:1000) |
| VPS15 | Novus | NBP1-30463 | Rabbit | WB (1:1000) |
| Atg14 | Cell Signaling | 96752 | Rabbit | WB (1:1000) |
| AMBRA1 | Novus | NB110-87318 | Rabbit | WB (1:1000) |
| UVRAG | Cell Signaling | 13115 | Rabbit | WB (1:1000) |
| HA | Cell Signaling | 3724 | Rabbit | WB (1:5000) |
| FLAG | GeneTex | GTX115043 | Rabbit | WB (1:5000) |
| γH2A.X | Sigma-Aldrich | 05-636 | Mouse | WB (1:1000) |
| H3 | Abcam | ab1791 | Rabbit | WB (1:1000) |
| p62/SQSTM1 | Novus | NBP1-48320 | Rabbit | IF (1:300) |
| Caspase 3 | Abcam | ab32351 | Rabbit | WB (1:1000) |
| PARP | Santa Cruz | sc-8007 | Mouse | WB (1:1000) |
| Ubiquitin | Abcam | Ab7254 | Mouse | WB (1:1000) |
| Ubiquitin (FK2) | Enzo | BML-PW8810-0100 | Mouse | IF (1:300) |
| p-Smad2 | Cell Signaling | 8828 | Rabbit | WB (1:1000) |
| Smad2 | Cell Signaling | 8685 | Rabbit | WB (1:1000) |

| **Table S2: Sequences of PCR primers** | | |
| --- | --- | --- |
| Primers | Forward (5’→3’) | Reverse (5’→3’) |
| *BCRP3* | CACAACCTCGCCACTGTCTT | CACTGGCATCTAGGCCTTCG |
| *GAPDH* | tgttgccatcaatgacccctt | ctccacgacgtactcagcg |
| *Neat1* | cagttagtttatcagttctCCcatcca | Gttgttgtcgtcacctttcaactct |
| *DAPK1* | CGAGGTGATGGTGTATGGTG | CTGTGCTTTGCTGGTGGA |
| *P15* | CACCCCCACCCACCTAATTC | TGAGTGTCGAGGGCCAGATA |
| *P21* | CCTCATCCCGTGTTCTCCTTT | GTACCACCCAGCGGACAAGT |

| **Table S3: List of proteins accumulated in *BCRP3*-deficient cells under proteotoxicity** | | | |
| --- | --- | --- | --- |
| **Accession** | **Entry name** | **Abundance Ratio** | **Adj. P-Value** |
| JUND | P17535 | 100 | 2.56612E-16 |
| CDKN1B | [P46527](https://www.uniprot.org/uniprot/P46527) | 100 | 2.56612E-16 |
| MRPS24 | [Q96EL2](https://www.uniprot.org/uniprot/Q96EL2) | 100 | 2.56612E-16 |
| ATAD2B | [Q9ULI0](https://www.uniprot.org/uniprot/Q9ULI0) | 100 | 2.56612E-16 |
| NAV1 | [Q8NEY1](https://www.uniprot.org/uniprot/Q8NEY1) | 4.857 | 4.94107E-10 |
| TLK2 | [Q86UE8](https://www.uniprot.org/uniprot/Q86UE8) | 15.023 | 2.56612E-16 |
| GALM | [Q96C23](https://www.uniprot.org/uniprot/Q96C23) | 100 | 2.56612E-16 |
| CCDC33 | [Q8N5R6](https://www.uniprot.org/uniprot/Q8N5R6) | 6.432 | 2.55111E-12 |
| MRPL34 | [Q9BQ48](https://www.uniprot.org/uniprot/Q9BQ48) | 100 | 2.56612E-16 |
| RAF1 | [P04049](https://www.uniprot.org/uniprot/P04049) | 9.954 | 2.56612E-16 |
| METTL17 | [Q9H7H0](https://www.uniprot.org/uniprot/Q9H7H0) | 10.183 | 2.56612E-16 |
| DNAJC19 | [Q96DA6](https://www.uniprot.org/uniprot/Q96DA6) | 100 | 2.56612E-16 |
| FAM207A | [Q9NSI2](https://www.uniprot.org/uniprot/Q9NSI2) | 100 | 2.56612E-16 |
| CIZ1 | [Q9ULV3](https://www.uniprot.org/uniprot/Q9ULV3) | 100 | 2.56612E-16 |
| RBX1 | [P62877](https://www.uniprot.org/uniprot/P62877) | 2.088 | 1.76306E-08 |
| CHMP6 | [Q96FZ7](https://www.uniprot.org/uniprot/Q96FZ7) | 100 | 2.56612E-16 |
| SIX2 | [Q9NPC8](https://www.uniprot.org/uniprot/Q9NPC8) | 100 | 2.56612E-16 |
| MAFK | [O60675](https://www.uniprot.org/uniprot/O60675) | 17.448 | 2.56612E-16 |
| MPLKIP | [Q8TAP9](https://www.uniprot.org/uniprot/Q8TAP9) | 15.132 | 2.56612E-16 |
| CLU | [P10909](https://www.uniprot.org/uniprot/P10909) | 1.566 | 2.84342E-05 |
| EIF4E2 | [O60573](https://www.uniprot.org/uniprot/O60573) | 100 | 2.56612E-16 |
| ERBB2 | [P04626](https://www.uniprot.org/uniprot/P04626) | 100 | 2.56612E-16 |
| DTX3L | [Q8TDB6](https://www.uniprot.org/uniprot/Q8TDB6) | 11.1 | 2.56612E-16 |
| ATM | [Q13315](https://www.uniprot.org/uniprot/Q13315) | 9.922 | 2.56612E-16 |
| SLCO2A1 | [Q92959](https://www.uniprot.org/uniprot/Q92959) | 11.85 | 2.56612E-16 |
| MCRIP2 | [Q9BUT9](https://www.uniprot.org/uniprot/Q9BUT9) | 100 | 2.56612E-16 |
| SVIL | [O95425](https://www.uniprot.org/uniprot/O95425) | 9.626 | 2.56612E-16 |
| TSC22D4 | [Q9Y3Q8](https://www.uniprot.org/uniprot/Q9Y3Q8) | 2.143 | 0.012880251 |
| LEPROTL1 | [O95214](https://www.uniprot.org/uniprot/O95214) | 12.523 | 2.56612E-16 |
| ARNT | [P27540](https://www.uniprot.org/uniprot/P27540) | 100 | 2.56612E-16 |
| CRTC1 | [Q6UUV9](https://www.uniprot.org/uniprot/Q6UUV9) | 100 | 2.56612E-16 |
| CRELD2 | [Q6UXH1](https://www.uniprot.org/uniprot/Q6UXH1) | 1.668 | 0.000329277 |
| RFX1 | [P22670](https://www.uniprot.org/uniprot/P22670) | 8.366 | 2.56612E-16 |
| TGFB1 | [P01137](https://www.uniprot.org/uniprot/P01137) | 100 | 2.56612E-16 |
| ALDH6A1 | [Q02252](https://www.uniprot.org/uniprot/Q02252) | 2.765 | 0.000212194 |
| THEM4 | [Q5T1C6](https://www.uniprot.org/uniprot/Q5T1C6) | 100 | 2.56612E-16 |
| FIS1 | [Q9Y3D6](https://www.uniprot.org/uniprot/Q9Y3D6) | 1.652 | 0.000103184 |
| PSMA6 | [P60900](https://www.uniprot.org/uniprot/P60900) | 1.505 | 1.43724E-08 |
| MED29 | [Q9NX70](https://www.uniprot.org/uniprot/Q9NX70) | 100 | 2.56612E-16 |
| FLG2 | [Q5D862](https://www.uniprot.org/uniprot/Q5D862) | 1.704 | 0.022994832 |
| MND1 | [Q9BWT6](https://www.uniprot.org/uniprot/Q9BWT6) | 100 | 2.56612E-16 |
| CEP44 | [Q9C0F1](https://www.uniprot.org/uniprot/Q9C0F1) | 100 | 2.56612E-16 |
| NDUFB4 | [O95168](https://www.uniprot.org/uniprot/O95168) | 1.597 | 0.001565727 |
| AGMAT | [Q9BSE5](https://www.uniprot.org/uniprot/Q9BSE5) | 100 | 2.56612E-16 |
| HLA-A | [P04439](https://www.uniprot.org/uniprot/P04439) | 1.583 | 0.024314864 |
| TBL1XR1 | [Q9BZK7](https://www.uniprot.org/uniprot/Q9BZK7) | 1.569 | 1.38554E-05 |
| SAMD9 | [Q5K651](https://www.uniprot.org/uniprot/Q5K651) | 100 | 2.56612E-16 |
| HIST1H1C | [P16403](https://www.uniprot.org/uniprot/P16403) | 100 | 2.56612E-16 |
| LACTB | [P83111](https://www.uniprot.org/uniprot/P83111) | 100 | 2.56612E-16 |
| QSOX2 | [Q6ZRP7](https://www.uniprot.org/uniprot/Q6ZRP7) | 1.618 | 0.021114422 |
| SHPK | [Q9UHJ6](https://www.uniprot.org/uniprot/Q9UHJ6) | 1.661 | 0.000650569 |
| CENPM | [Q9NSP4](https://www.uniprot.org/uniprot/Q9NSP4) | 2.382 | 2.25898E-07 |
| CEP72 | [Q9P209](https://www.uniprot.org/uniprot/Q9P209) | 100 | 2.56612E-16 |
| RPAP2 | [Q8IXW5](https://www.uniprot.org/uniprot/Q8IXW5) | 100 | 2.56612E-16 |
| BECN1 | [Q14457](https://www.uniprot.org/uniprot/Q14457) | 100 | 2.56612E-16 |
| DSP | [P15924](https://www.uniprot.org/uniprot/P15924) | 1.634 | 0.000629904 |
| FAM20B | [O75063](https://www.uniprot.org/uniprot/O75063) | 2.659 | 0.000356707 |
| CASP3 | [P42574](https://www.uniprot.org/uniprot/P42574) | 100 | 2.56612E-16 |
| DNPH1 | [O43598](https://www.uniprot.org/uniprot/O43598) | 1.524 | 0.001889524 |
| PCDHGA12 | [O60330](https://www.uniprot.org/uniprot/O60330) | 2.804 | 2.10301E-05 |
| CDC42SE2 | [Q9NRR3](https://www.uniprot.org/uniprot/Q9NRR3) | 100 | 2.56612E-16 |
| TRAPPC12 | [Q8WVT3](https://www.uniprot.org/uniprot/Q8WVT3) | 100 | 2.56612E-16 |
| OR2AG1 | [Q9H205](https://www.uniprot.org/uniprot/Q9H205) | 1.745 | 1.9695E-07 |
| B4GAT1 | [O43505](https://www.uniprot.org/uniprot/O43505) | 9.671 | 2.56612E-16 |
| MVP | [Q14764](https://www.uniprot.org/uniprot/Q14764) | 1.531 | 0.005032874 |
| GAMT | [Q14353](https://www.uniprot.org/uniprot/Q14353) | 1.508 | 0.00598117 |
| RNASET2 | [O00584](https://www.uniprot.org/uniprot/O00584) | 2.451 | 0.000845639 |
| SLC7A6OS | [Q96CW6](https://www.uniprot.org/uniprot/Q96CW6) | 9.418 | 2.56612E-16 |
| OR1L3 | [Q8NH93](https://www.uniprot.org/uniprot/Q8NH93) | 2.089 | 0.020544957 |
| OXNAD1 | [Q96HP4](https://www.uniprot.org/uniprot/Q96HP4) | 100 | 2.56612E-16 |
| SLC33A1 | [O00400](https://www.uniprot.org/uniprot/O00400) | 14.663 | 2.56612E-16 |
| PARL | [Q9H300](https://www.uniprot.org/uniprot/Q9H300) | 12.579 | 2.56612E-16 |
| SIRT1 | [Q96EB6](https://www.uniprot.org/uniprot/Q96EB6) | 10.099 | 2.56612E-16 |
| SOD2 | [P04179](https://www.uniprot.org/uniprot/P04179) | 1.783 | 2.50794E-05 |
| NAP1L5 | [Q96NT1](https://www.uniprot.org/uniprot/Q96NT1) | 1.741 | 0.045348676 |
| MTFP1 | [Q9UDX5](https://www.uniprot.org/uniprot/Q9UDX5) | 1.928 | 0.045363831 |
| STARD7 | [Q9NQZ5](https://www.uniprot.org/uniprot/Q9NQZ5) | 8.571 | 2.56612E-16 |
| POLR2M | [P0CAP2](https://www.uniprot.org/uniprot/P0CAP2) | 1.538 | 0.007524455 |
| HCP5 | [Q6MZN7](https://www.uniprot.org/uniprot/Q6MZN7) | 10.195 | 2.56612E-16 |
| PTGES | [O14684](https://www.uniprot.org/uniprot/O14684) | 100 | 2.56612E-16 |
| TTC4 | [O95801](https://www.uniprot.org/uniprot/O95801) | 1.637 | 0.001134109 |
| OCA2 | [Q04671](https://www.uniprot.org/uniprot/Q04671) | 2.069 | 0.001053609 |
| KATNB1 | [Q9BVA0](https://www.uniprot.org/uniprot/Q9BVA0) | 100 | 2.56612E-16 |
| S100A3 | [P33764](https://www.uniprot.org/uniprot/P33764) | 1.614 | 0.030430704 |
| TRIM24 | [O15164](https://www.uniprot.org/uniprot/O15164) | 8.375 | 2.56612E-16 |
| CPM | [P14384](https://www.uniprot.org/uniprot/P14384) | 100 | 2.56612E-16 |
| TCIRG1 | [Q13488](https://www.uniprot.org/uniprot/Q13488) | 1.617 | 0.012880251 |
| TMED8 | [Q6PL24](https://www.uniprot.org/uniprot/Q6PL24) | 100 | 2.56612E-16 |
| TOR1B | [O14657](https://www.uniprot.org/uniprot/O14657) | 2.081 | 0.025207581 |
| ANKRD50 | [Q9ULJ7](https://www.uniprot.org/uniprot/Q9ULJ7) | 100 | 2.56612E-16 |
| SMAD2 | [Q15796](https://www.uniprot.org/uniprot/Q15796) | 100 | 2.56612E-16 |
| ARAP3 | [Q8WWN8](https://www.uniprot.org/uniprot/Q8WWN8) | 2.057 | 0.005203143 |
| KDELR2 | [P33947](https://www.uniprot.org/uniprot/P33947) | 2.235 | 0.002599044 |
| KRT2 | [P35908](https://www.uniprot.org/uniprot/P35908) | 1.589 | 1.06202E-08 |
| KRT5 | [P13647](https://www.uniprot.org/uniprot/P13647) | 2.511 | 0.000176187 |
| MT-ND1 | [P03886](https://www.uniprot.org/uniprot/P03886) | 1.995 | 0.018851536 |
| CDKN2AIPNL | [Q96HQ2](https://www.uniprot.org/uniprot/Q96HQ2) | 11.233 | 2.56612E-16 |
| GABARAP | [O95166](https://www.uniprot.org/uniprot/O95166) | 1.856 | 0.001519523 |
| SUDS3 | [Q9H7L9](https://www.uniprot.org/uniprot/Q9H7L9) | 100 | 2.56612E-16 |
| KRT14 | [P02533](https://www.uniprot.org/uniprot/P02533) | 2.711 | 3.39108E-14 |
| ELP5 | [Q8TE02](https://www.uniprot.org/uniprot/Q8TE02) | 100 | 2.56612E-16 |
| VMA21 | [Q3ZAQ7](https://www.uniprot.org/uniprot/Q3ZAQ7) | 1.887 | 6.82074E-07 |
| JADE1 | [Q6IE81](https://www.uniprot.org/uniprot/Q6IE81) | 2.18 | 0.022905035 |
| SPG7 | [Q9UQ90](https://www.uniprot.org/uniprot/Q9UQ90) | 100 | 2.56612E-16 |
| PABPC3 | [Q9H361](https://www.uniprot.org/uniprot/Q9H361) | 9.963 | 2.56612E-16 |
| HEMGN | [Q9BXL5](https://www.uniprot.org/uniprot/Q9BXL5) | 11.545 | 2.56612E-16 |
| ASAP2 | [O43150](https://www.uniprot.org/uniprot/O43150) | 2.145 | 2.83739E-14 |
| FUCA2 | [Q9BTY2](https://www.uniprot.org/uniprot/Q9BTY2) | 100 | 2.56612E-16 |
| PNPLA8 | [Q9NP80](https://www.uniprot.org/uniprot/Q9NP80) | 9.997 | 2.56612E-16 |
| SDSL | [Q96GA7](https://www.uniprot.org/uniprot/Q96GA7) | 100 | 2.56612E-16 |
| CELSR3 | [Q9NYQ7](https://www.uniprot.org/uniprot/Q9NYQ7) | 100 | 2.56612E-16 |
| KRT73 | [Q86Y46](https://www.uniprot.org/uniprot/Q86Y46) | 100 | 2.56612E-16 |
| RALGAPA1 | [Q6GYQ0](https://www.uniprot.org/uniprot/Q6GYQ0) | 100 | 2.56612E-16 |
| PHKA1 | [P46020](https://www.uniprot.org/uniprot/P46020) | 100 | 2.56612E-16 |
| NSMCE4A | [Q9NXX6](https://www.uniprot.org/uniprot/Q9NXX6) | 100 | 2.56612E-16 |
| PRR11 | [Q96HE9](https://www.uniprot.org/uniprot/Q96HE9) | 100 | 2.56612E-16 |
| GPN2 | [Q9H9Y4](https://www.uniprot.org/uniprot/Q9H9Y4) | 100 | 2.56612E-16 |
| LEMD2 | [Q8NC56](https://www.uniprot.org/uniprot/Q8NC56) | 100 | 2.56612E-16 |
| METTL26 | [Q96S19](https://www.uniprot.org/uniprot/Q96S19) | 100 | 2.56612E-16 |
| ZDHHC4 | [Q9NPG8](https://www.uniprot.org/uniprot/Q9NPG8) | 100 | 2.56612E-16 |
| ASPSCR1 | [Q9BZE9](https://www.uniprot.org/uniprot/Q9BZE9) | 100 | 2.56612E-16 |
| PTTG1 | [O95997](https://www.uniprot.org/uniprot/O95997) | 100 | 2.56612E-16 |
| FOXP1 | [Q9H334](https://www.uniprot.org/uniprot/Q9H334) | 100 | 2.56612E-16 |
| YEATS4 | [O95619](https://www.uniprot.org/uniprot/O95619) | 100 | 2.56612E-16 |
| KIF3B | [O15066](https://www.uniprot.org/uniprot/O15066) | 100 | 2.56612E-16 |
| USP22 | [Q9UPT9](https://www.uniprot.org/uniprot/Q9UPT9) | 100 | 2.56612E-16 |
| EXOC6B | [Q9Y2D4](https://www.uniprot.org/uniprot/Q9Y2D4) | 100 | 2.56612E-16 |
| PHF2 | [O75151](https://www.uniprot.org/uniprot/O75151) | 100 | 2.56612E-16 |
| ERC2 | [O15083](https://www.uniprot.org/uniprot/O15083) | 10.412 | 2.56612E-16 |
| RFXAP | [O00287](https://www.uniprot.org/uniprot/O00287) | 11.752 | 2.56612E-16 |
| RAD51 | [Q06609](https://www.uniprot.org/uniprot/Q06609) | 100 | 2.56612E-16 |
| SIKE1 | [Q9BRV8](https://www.uniprot.org/uniprot/Q9BRV8) | 100 | 2.56612E-16 |
| SCN4A | [P35499](https://www.uniprot.org/uniprot/P35499) | 14.319 | 2.56612E-16 |
| POLG | [P54098](https://www.uniprot.org/uniprot/P54098) | 100 | 2.56612E-16 |
